# Early Modern Humans and Morphological Variation in Southeast Asia: Fossil Evidence from Tam Pa Ling, Laos

**DOI:** 10.1371/journal.pone.0121193

**Published:** 2015-04-07

**Authors:** Fabrice Demeter, Laura Shackelford, Kira Westaway, Philippe Duringer, Anne-Marie Bacon, Jean-Luc Ponche, Xiujie Wu, Thongsa Sayavongkhamdy, Jian-Xin Zhao, Lani Barnes, Marc Boyon, Phonephanh Sichanthongtip, Frank Sénégas, Anne-Marie Karpoff, Elise Patole-Edoumba, Yves Coppens, José Braga

**Affiliations:** 1 Département Homme Nature Société, Unité Mixte de Recherche 7206, Unité Scientifique du Muséum 104, Muséum national d’Histoire naturelle, Musée de l’Homme, Paris, France; 2 Chaire Sorbonne Université FaciLe, Institut du Calcul et de la Simulation, Université Pierre et Marie Curie, Paris, France; 3 Department of Anthropology, University of Illinois at Urbana-Champaign, Urbana, Illinois, United States of America; 4 Department of Environment and Geography, Macquarie University, Sydney, Australia; 5 Ecole et Observatoire des Sciences de la Terre, Institut de Physique du Globe de Strasbourg, Centre National de la Recherche Scientifique, Unité mixte de recherche 7516, Université de Strasbourg, Strasbourg, France; 6 Unité Propre de Recherche 2147, Centre National de la Recherche Scientifique, Paris, France; 7 Institute of Vertebrate Paleontology and Paleoanthropology, Chinese Academy of Sciences, Beijing, China; 8 Department of National Heritage, Ministry of Information and Culture, Vientiane, LAO People’s Democratic Republic; 9 School of Earth Sciences, Steele Building, University of Queensland, Brisbane, Australia; 10 Institut de Chimie et Procédés pour l’Energie, l’Environnement et la Santé, Université de Strasbourg, Centre National de la Recherche Scientifique, Unité Mixte de Recherche 7515 and Ecole et Observatoire des Sciences de la Terre, Unité Mixte de Recherche 7516 and 7362, Strasbourg, France; 11 Muséum d’Histoire Naturelle de la Rochelle, La Rochelle, France; 12 Collège de France, Paris, France; 13 Unité Mixte de Recherche 5288 Anthropobiologie et Imagerie Anatomique, Centre National de la Recherche Scientifique, Université Paul Sabatier, Toulouse, France; 14 Evolutionary Studies Institute and Kromdraai Research Project, University of the Witwatersrand, Johannesburg, South Africa; Université de Poitiers, FRANCE

## Abstract

Little is known about the timing of modern human emergence and occupation in Eastern Eurasia. However a rapid migration out of Africa into Southeast Asia by at least 60 ka is supported by archaeological, paleogenetic and paleoanthropological data. Recent discoveries in Laos, a modern human cranium (TPL1) from Tam Pa Ling‘s cave, provided the first evidence for the presence of early modern humans in mainland Southeast Asia by 63-46 ka. In the current study, a complete human mandible representing a second individual, TPL 2, is described using discrete traits and geometric morphometrics with an emphasis on determining its population affinity. The TPL2 mandible has a chin and other discrete traits consistent with early modern humans, but it retains a robust lateral corpus and internal corporal morphology typical of archaic humans across the Old World. The mosaic morphology of TPL2 and the fully modern human morphology of TPL1 suggest that a large range of morphological variation was present in early modern human populations residing in the eastern Eurasia by MIS 3.

## Introduction

In December 2009, a partial human cranium with fully modern morphology (TPL1) was recovered from Tam Pa Ling, Laos [1]. In December 2010, a complete human mandible (TPL2) was recovered from the same site. Based on non-alignment between the maxilla of the TPL1 cranium and the TPL2 mandible, these remains represent two separate individuals. These fossils are the first Pleistocene human remains discovered in Laos since 1934 [2, 3], and they establish the definitive presence of early modern humans in mainland Southeast Asia by a minimum of 46 ka and likely as much as 20,000 years earlier [1].

Tam Pa Ling is one of only a handful of well-dated, early modern human fossil sites in eastern Asia and Australasia. The partial skeleton from Tianyuandong, northern China, is dated to ~ 40 ka [4], as is the partial cranium from Laibin, southern China and the Niah 1 cranium from Sarawak, Malaysia [5–7]. Other fossils such as Liujiang and Ziyang from China may be as old, but their provenance is uncertain [8–13]. The oldest modern human remains from south Asia at Fa Hein in Sri Lanka are modestly younger at ~36 ka cal BP [14, 15]. Modern human fossils are present in Australia by at least 40 ka though the timing of the earliest remains is uncertain [16]. Fossils from Callao Cave, Luzon, Philippines have been dated to ~ 67 ka, although their specific attribution is unclear [17]. A partial mandible from Zhirendong, southern China, which is dated to ~ 100 ka, shows a mixture of archaic and modern human morphology [18], late archaic humans fossils from the Chinese sites of Maba and Xujiayao are dated to 125–69 ka [9, 10, 19–21], while a newly discovered archaic *Homo* mandible from the Taiwanese site of Penghu is dated to 190-130/70-10 ka [52]. This slowly accumulating record of human fossils from the Late Pleistocene of Eastern Eurasia lends additional data to questions about modern human origins at the eastern periphery of the Old World.

The purpose of the current analysis is first to strengthen and extend the chronological framework for the TPL deposits with additional dated geological samples. Secondly, TPL2 is described with an emphasis on its mosaic morphology. Its affinity is determined on the basis of discrete traits and geometric morphometrics in order to place the fossil in its appropriate evolutionary context and to determine the position of this site, northern Laos and the Southeast Asian mainland more generally in Late Pleistocene human evolution.

### Context and dating

**Fig 1:**
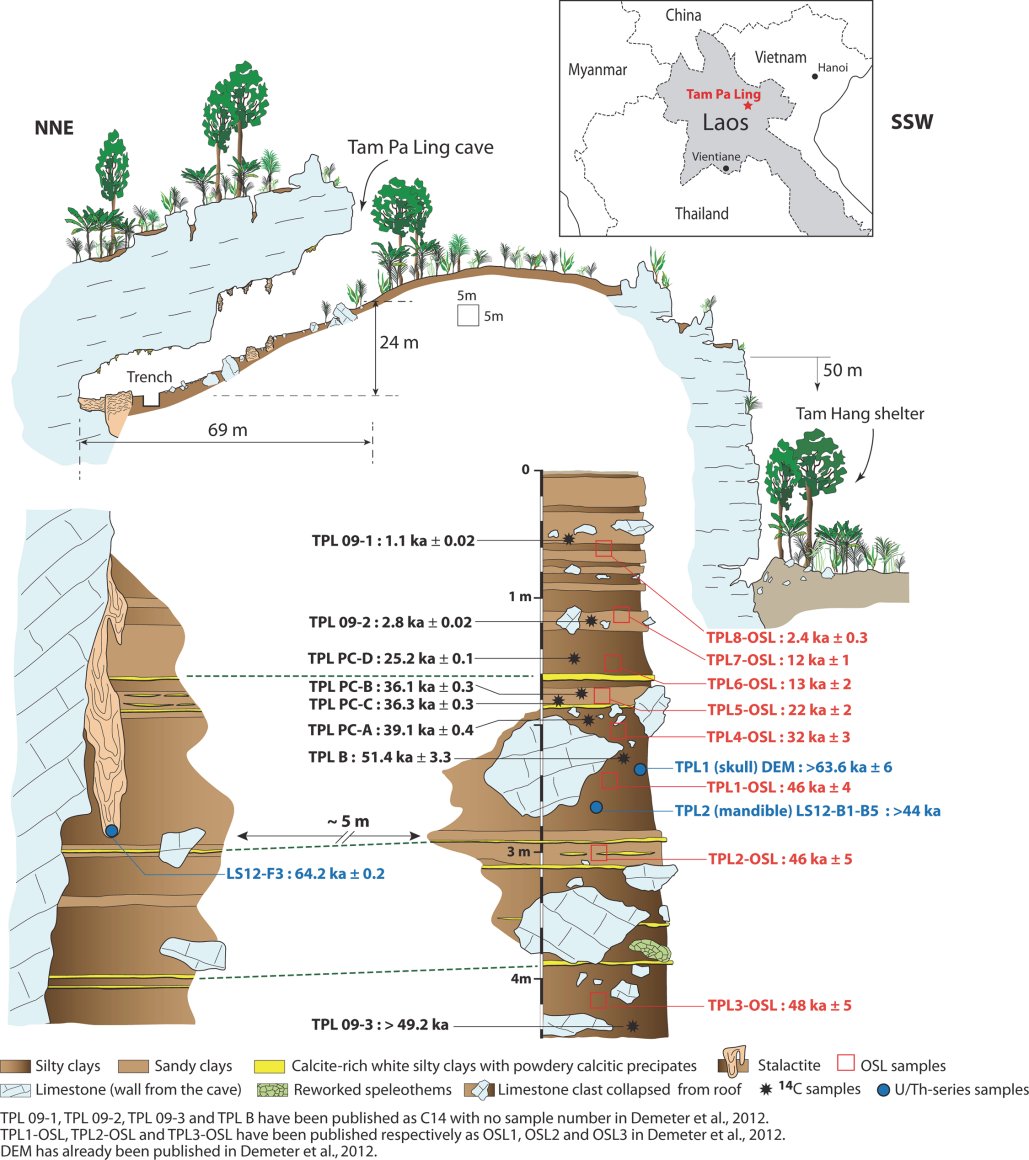
Site of Tam Pa Ling, Laos. TPL is located on the upper plateau of the Pa Hang Mountain with the Tam Hang rock shelter at the mountain’s base. The 4.5 m stratigraphic section shows the accumulation of sandy and silty clay layers punctuated by seven powdery, calcitic precipitates from the TPL trench. Provenance of the charcoals sampled for ^14^C dating and soil sampled for OSL and TL dating is identified on the stratigraphy. TPL1 was recovered at a depth of 2.35 m; TPL2 was found at a depth of 2.65 m. Inset: Location of TPL in Huà Pan Province, Laos.

Tam Pa Ling is located in Huà Pan Province, Laos, approximately 260 km NNE of Vientiane (20°12’31.4” N, 103°24’35.2”E, elev. 1,170 m). The cave is part of the Annamite Mountains, which straddle the Laos-Vietnam border (Fig 1). The landscape consists of tower karsts derived from the dissolution of Upper Carboniferous to Permian limestone beds, with a dense network of caves and galleries. Tam Pa Ling has a single, south-facing opening that descends 65 m to the main gallery. This gallery measures 30 m in length along a north-south axis and 40 m in width along an east-west axis. The TPL excavation is located at the east end of the gallery at the base of the sloped entrance (S1 and S2 Figs). Sediments at the base of the slope represent periodic, slopewash deposition from the argillaceous-dominated bank at the entrance of the cave, and the stratigraphic integrity of these layers has been established [1].

**Fig 2:**
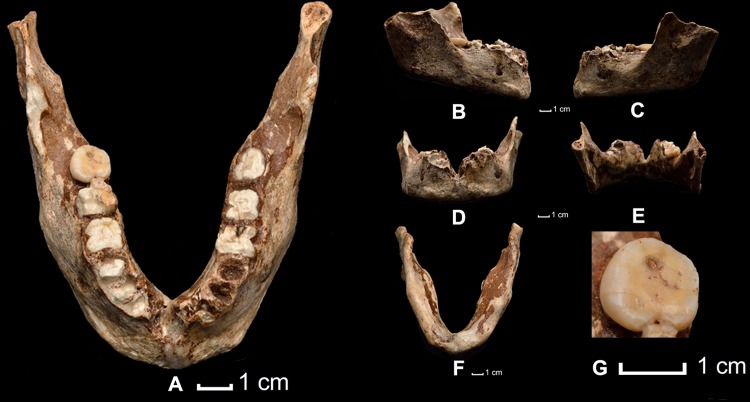
Human fossil remains designated as TPL2. (A) Mandible in *norma verticalis*; (B) mandible in *norma lateralis*, right side; (C) mandible in *norma latelaris*, left side; (D) mandible in *norma facialis* external; (E) mandible in *norma facialis* internal (F) mandible in *norma basilaris*; (G) occlusal view of the right M_3_.

The site was discovered in 2009 and has been excavated since December 2010. In December 2010, cranial remains of a single, modern human were found at a depth of 2.35 m (TPL1, S3 Fig) [1]. Analysis of these remains revealed completely modern anatomy with no archaic features. In December 2011, a complete human mandible (TPL2) was recovered at a depth of 2.65 m and approximately one meter from the source of the TPL1 cranium (Fig 2, and S4, S5 Figs). It was broken at the symphyseal plane, and the right and left halves were recovered approximately 20 cm apart. Despite their proximity in the excavation, TPL1 and TPL2 represent two separate individuals based on differences in size and morphology, non-alignment between the maxilla of the TPL1 cranium and the TPL2 mandible and the difference in the degree of occlusal attrition of the TPL1 teeth and the TPL2 M_3_. No artifacts have been found at the site, and there is no evidence of an occupation surface within the stratigraphic section or within the cave. As is the case with TPL1, the source of the fossil is unknown but the state of preservation and the absence of water-rolling evidence suggest that it originated at or near the entrance of the cave and was subsequently carried into the cave via slopewash transport and buried within the cave stratigraphy.

Micromammal and reptile remains were recovered in the TPL trench from 0.74 to 4.5 m in depth. Preliminary analysis reveals that the rodent material is dominated by three taxa, *Leopoldamys* cf. *sabanus*, *Niviventer* sp. and *Rattus* sp. (77% of the identifiable remains), with the remaining material comprised of *Berylmys* sp., *Chiropodomys* sp., *Hapalomys* sp., *Belomys pearsonii* and some unidentified Arvicolinae (S1 Text and S9 Table). Most of these taxa are still present today in the TPL region, except for *Belomys pearsonii* and the Arvicolinae. The latter taxon might indicate a cooler environmental component.

The stratigraphy of the site has been described elsewhere [1] and is described in detail in S1 Text. As previously reported, luminescence ages for the surrounding sediments provided a minimum age of 46 ka for sedimentary deposition and the associated fossils, and direct U/Th-series dating of the frontal bone provided a minimum age for bone formation of >63 ka. Radiocarbon results supported this antiquity but were outside the generally accepted age range for this method. Despite this, the chronology has been contested [22] with questions raised by a potential sedimentary hiatus of ~ 44 ka in the upper stratigraphy. Therefore, in 2012, additional OSL, U/Th-series and radiocarbon samples were collected throughout the 4.5 m section to extend the chronology, confirm the integrity of the deposits and reduce the age range of the unknown hiatus period (see S1 Text for dating strategy, sampling locations and sample characteristics). In addition, a bone fragment from the left condyle of TPL2 was sampled for U/Th-series dating to confirm the dating of the fossil itself.

The extended OSL chronology agrees with and builds on the previous chronology (Fig 1 and Tables 1, 2; S1 Text and S7 Table). The sedimentary “hiatus” of ~ 44 ka that was previously identified at the top of the stratigraphic column is reduced to ~10 ka between samples TPL4-OSL and TPL5-OSL and ~ 9 ka between samples TPL5-OSL and TPL6-OSL. Both of these sections are separated by a layer of powdery calcite precipitate (‘moonmilk’), which may represent a cessation of sedimentation in the cave or an arrival of calcium-rich water. Between samples TPL1-OSL and TPL4-OSL, and between TPL7-OSL and TPL8-OSL there are hiatuses of ~ 14 ka and 10 ka, respectively, which represent the largest gaps in sedimentary accumulation with no obvious record of a break in the stratigraphy (see S1 Text). However, in both of these locations there is a large amount of rockfall that may represent a period of collapse and the temporary blockage of the cave from allochthonous sedimentary inputs.

**Table 1:** OSL single-grain dating of sediments from Tam Pa Ling: dose rate data, equivalent doses and ages.

Sample code	Depth	Grain size	Beta dose rate^a^	Field gamma dose	Cosmic-ray dose	Water content^d^	Total dose	Stat. model^f^	Equiv. dose^g^ ^,^ ^h^	Age^i^
				rate^b^	rate^c^		rate^e^		(Gy)	(ka)
	(m)	(μm)	(Gy ka^-1^)	(Gy ka^-1^)	(Gy ka^-1^)	(%)	(Gy ka^-1^)			
TPL8-OSL	0.60	180–212	1.295 ± 0.064	0.852 ± 0.04	0.015	28 / 20 ± 5	2.51 ± 0.14	MAM	6 ± 1	2.4 ± 0.3
TPL7-OSL	1.20	180–212	1.143 ± 0.057	0.436 ± 0.05	0.014	38 / 30 ± 5	1.63 ± 0.10	MAM	20 ± 1	12 ± 1
TPL6-OSL	1.60	180–212	1.295 ± 0.062	0.844 ± 0.05	0.014	47 / 30 ± 10	2.19 ± 0.22	MAM	29 ± 3	13 ± 2
TPL5-OSL	1.85	180–212	1.393 ± 0.063	1.008 ± 0.04	0.015	31 / 25 ± 5	2.45 ± 0.13	MAM	54 ± 4	22 ± 2
TPL4-OSL	2.10	180–212	1.513 ± 0.067	1.108 ± 0.04	0.015	30 / 25 ± 5	2.67 ± 0.16	MAM	86 ± 5	32 ± 3

^a^ Determined from beta counter measurements of dried and powdered sediment samples.

^b^ Determined from U, Th and K concentrations measured using a portable gamma-ray spectrometer at field water content

^c^ Time-averaged cosmic-ray dose rates (for dry samples), each assigned an uncertainty of ± 10%.

^d^ Field / time-averaged water contents, expressed as (mass of water/mass of dry sample) x 100. The latter values were used to calculate the total dose rates and OSL ages

^e^ Mean ± total (1σ) uncertainty, calculated as the quadratic sum of the random and systematic uncertainties. An internal dose rate of 0.03 Gy ka-1 is also included

^f^ Statistical model used to determine the dose distribution between grains-MAM—Minimum Age Model

^g^ Palaeodoses include a ± 2% systematic uncertainty associated with laboratory beta-source calibrations

^h^ OSL signal measured using single-grains of quartz—between 800–1800 grains were run for each sample with between 6–20% of the grains emitting an acceptable luminescence signal, with the De derived from a MAM.

^i^ Uncertainties at 68% confidence interval

**Table 2:** Uranium-series dating of Tam Pa Ling stalactite and TPL2 bone: ages and supporting data.

Sample Name	Sample depth (m)^a^ ^,^ ^b^	U (ppm)	^232^Th (ppb)	(^230^Th/ ^232^Th) ratio^c^	(^230^Th/^238^U) ratio	(^234^U/ ^238^U) ratio^c^	Uncorr. Age (ka)^c^	Corr. Age (ka)^c^	Corr. Initial (^234^U/ ^238^U)
LS12-B1	2.6	67.20	6932	12.19	0.414 ± 0.002	1.38	38.4 ± 0.2	36.2 ± 1.0	1.43
LS12-B2	2.6	55.42	1196	66.02	0.469 ± 0.002	1.39	44.1 ± 0.2	43.7 ± 0.3	1.44
LS12-B3	2.6	68.21	3942	21.84	0.416 ± 0.001	1.39	38.2 ± 0.1	37.0 ± 0.5	1.44
LS12-B4	2.6	61.26	3591	21.22	0.410 ± 0.001	1.38	37.7 ± 0.1	36.5 ± 0.5	1.43
LS12-B5	2.6	57.19	3147	25.37	0.460 ± 0.002	1.39	43.0 ± 0.2	41.8 ± 0.5	1.45
LS12-F3	2.8	0.24	5.13	128.69	0.908 ± 0.002	1.96	64.2 ± 0.2	64.0 ± 0.2	2.16

^a^ Measured from base of the cave floor to sampling height

^b^ Depth of the bone in the sediment column

^c^ Uncertainties at 95% confidence interval.

The OSL age estimates (TPL4-8-OSL, Fig 1 and Table 1) display a steady increase in age with depth and are stratigraphically consistent over the 4.5 m of excavation but show little agreement with the calibrated radiocarbon results within known limitations of the techniques. The radiocarbon results are generally older than the timing of sedimentary deposition according to the OSL chronology. As the burning that created the charcoal that was used for the radiocarbon chronology did not occur *in situ* there is a strong likelihood that the charcoal represents old carbon that was washed into the cave (from natural or anthropogenic fires) (Fig 1 and S7 Table). While the radiocarbon dates may be useful for displaying the antiquity of the deposits, they are not reliable for establishing the timing of fossil deposition. Thus the radiocarbon chronology is consistently older than the OSL chronology throughout the upper section and has been presented to demonstrate the problems linked with radiocarbon dating in ‘sink’ or ‘wash-in’ (non-occupation) caves (see S1 Text).

Efforts to obtain calcite suitable for U/Th-series dating from the powdery moonmilk layers were unsuccessful, but the tip of an overhanging stalactite (LS12–F3, Table 2) corresponding to the level of the human cranium and mandible provided a useful maximum age of ~64 ka for sedimentary infilling at that depth (Fig 1). The U/Th-series dating of the TPL2 bone fragment (LS12-B1-B5) proved equally challenging and could not be microdrilled for U/Th-series profiling due to its porous nature. As such, the analysis was conducted on small handpicked fragments, which gave a minimum age for the fossil of ~44–36 ka. As the bone fragments used for U/Th-series dating are porous and contained inseparable, post-fossil, secondary calcite overgrowths, the individual fragment ages represent minimum ages for the fossil, i.e., the fossils cannot be any younger than ~44 ka, and should in fact be older. These age estimates are in agreement with the OSL burial ages within errors [1], but due to the sedimentary nature of the cave as a wash-in environment it is expected that the fossils were on the landscape for an unknown amount of time before being washed into the cave so should in fact be older than the depositional ages as suggested by the initial dating of TPL1 [1]. However, these new U/Th results still place a useful minimum constraint on the age of the fossils themselves, implying that they cannot be Holocene or last glacial maximum in age.

This new chronology confirms the validity of the previous chronological framework for the TPL1 cranium, confirms the integrity of the stratigraphic section and supports a greater antiquity for the TPL2 mandible than is suggested by the minimum age range of 44 to 36 ka.

## Results

### TPL2: Preservation

The mandible is largely complete with a well-preserved corpus that is broken at the symphyseal plane and the inferior right and left rami (Fig 2). The corpus has significant damage to the alveolar bone immediately surrounding the break at the midline symphysis, but otherwise shows only minor post-mortem scratching and abrasions. Despite damage to the alveolar bone at the midline break, significant details of the bone remained to refit the two halves in accurate anatomical position. The right ramus is broken at the level of the sigmoid notch and is missing the mandibular condyle and coronoid process. The left mandibular condyle is broken at the level of the sigmoid notch although the coronoid process is complete and the anterior portion of the sigmoid notch is present. A remaining portion of the condyle was used for U/Th-series dating (see “Context and dating”). Additional details about the preservation of the specimen and its reconstruction are provided in S1 Text.

The TPL2 mandible represents a mature adult individual, with complete formation of the corpus, alveolar bone, rami and condylar subchondral bone. Both third molars have erupted. The apices of all the roots, including those of the M_3_s, are completely closed (stage Ac^19^ as indicated on CT scans [27]). There is no evidence of antemortem tooth loss. All the teeth have been broken *post-mortem*, except M_3_, which shows occlusal attrition of grade 2 in the Molnar wear scale [28]. The combination of mandibular maturity, complete molar formation and occlusal wear on the M_3_ is consistent with an adult within the second half of the third decade. Sex of the individual is unknown.

The mandible has no pathological lesions. The right M_3_, the only preserved dental crown, shows moderate occlusal attrition and a small carious lesion on the distal occlusal surface.

### Overall dimensions

Linear and angular measurements and details of discrete traits of TPL2 are provided in S1, S2, and S3 Tables. The overall dimensions of TPL2 are small, with an inferior mandibular length of 77.0 mm and an estimated superior length of 87.0 mm, well below that of all other Pleistocene archaic or early modern human samples (Table 3). Mandibular breadth across the condyles cannot be assessed given their complete absence, but it is estimated to be between 92–100 mm. More reliably, bigonial breadth of TPL2 is 81.0 mm, and the dental arcade breadth at the M_2_ is 48.3 mm. There are no significant differences between any early modern human and archaic samples in estimated dental arcade breadth (Table 3), but TPL2 has a significantly smaller dental arcade breadth than all modern and archaic samples, including the closely contemporaneous mandible from Tianyuan cave (64.5 mm) or any other East Asian early modern humans (66.4 ± 2.2, n = 5) [29]. The only other *Homo* fossils that are similarly small in bigonial breadth and dental arcade breadth at the M_2_ are LB1 (83.0 mm (estimated) and 55.0 mm, respectively) and LB6 (71.0 mm and 53.0 mm, respectively) from Liang Bua, Flores [30].

**Table 3:** Overall mandibular and corpus dimensions of the TPL2 mandible and Late Pleistocene comparative samples (mean, standard deviation, N). See SI for fossils included in analyses. Parentheses indicate an estimated measurement.

	TPL 2	Archaic humans	East Asian modern humans	Western Eurasian modern humans
Superior length (mm)^a^	(87.0)	109.5*	98.7	102.2*
		6.5	2.2	5.7
		16	4	14
Arcade breadth at M_2_ (mm)	48.3	67.5*	66.4*	64.7*
		3.4	2.2	3.6
		16	5	12
Corpus height at mental foramen (M-69(1)) (mm)^b^	30.5 (R)/ 31.1 (L)	32.0	30.4	31.5
		3.6	1.5	4.2
		32	6	16
Corpus breadth at mental foramen (M-69(3) (mm)^b^	16.2 (R)/ 16.1 (L)	15.6	12.1*	12.6*
		1.8	0.73	1.5
		32	6	15
Robusticity index at MF^c^	1023.0	745.6*	543.0*	638.2*
		133.9	34.7	128.0
		16	5	9
Corpus breadth at M_1_/M_2_ (mm)	18.3 (R)/ 18.6 (L)	16.2	12.9*	14.2*
		1.6	1.2	1.8
		24	5	15

^a^ Midsagittal distance from the mid-condyles to infradentale.

^b^ M-#: Measurement definition in (23).

^c^ Corpus robusticity index at mental foramen = [(corpus height*corpus breadth)/arcade breadth at M_2_] *100 (30).

* Sample mean significantly different from TPL2 (α = 0.05 with multiple comparisons corrections).

### Modern human features: discrete traits

Due to post-mortem damage, the superior symphysis is unobservable; only the inferior half of the symphysis is visible (~ 16.4 mm from the basal margin). TPL2 has a clear chin, with a midline *tuber symphyseos* and paired lateral tubercles and a *mentum osseum* category rank 4 [18, 31–33]. This is the most common pattern demonstrated by modern humans, with 56.3% of East Asian early modern humans and a vast majority of western Eurasian early modern humans (71.4%) demonstrating this pattern. In contrast, 57.7% of Neandertals demonstrate a rank of 2 with no projecting *tuber symphyseos* and no archaic human has a value above a rank of 3 (S3 Table).

There is a single mental foramen on each side of the mandible, and each is located below P_4_-M_1_. In early modern humans, the mental foramen tends to have a mesial position (below P_4_) relative to a more distal position in archaic humans (below P_4_/M_1_ or M_1_) (Table 4). Although this is the “more archaic” condition, the position of the foramen in TPL2 is the same as that found in the majority of East Asian early modern humans (61.1%) (Table 4) and is most likely a reflection of the very short mandibular length of the individual [34, 35].

**Table 4:** Discrete traits of the TPL2 mandible and their frequency in Late Pleistocene comparative samples. See SI for fossils included in analyses.

	TPL2	Archaic humans	East Asian modern humans	Western Eurasian modern humans
Mentum osseum^1^	4	1 11.5%		3 2.9%
Rank %		2 57.7%	4 56.3%	4 71.4%
(N)		3 30.8%	5 43.8%	5 35.7%
		(26)	(8)	(35)
Mental foramen	P_4_ (R)			P_3_ 8.3%
Position %	M_1_ (L)	P_4_ 11.3%	P_4_ 27.8%	P_4_ 61.1%
(N)		P_4_/M_1_ 40.3%	P_4_/M_1_ 61.1%	P_4_/M_1_ 22.2%
		M_1_ 48.4%	M_1_ 11.1%	M_1_ 8.3%
		(31)	(9)	(36)
Retromolar space:	Absent	26.7 (30)	81.3 (8)	85.0 (30)
% absent (N)				
Mandibular notch symmetry:	Present	26.7 (14)	100 (5)	92.6 (27)
% present (N)				
Mandibular foramen:	Open	60.9 (23)	83.3 (6)	97.9 (24)
% open (N)				

^1^ Mentum osseum ranked on a 1–5 scale following [31].

In strict *norma lateralis*, the anteroinferior margin of the ramus crosses the alveolar plane at the distal neck of the M_3_ on the right side; although the left M_3_ crown is missing, the position of the anteroinferior margin of the left ramus is in approximately the same place. The roots of the rami lie above the lateral eminences, adjacent to the mesial M_3_s. A retromolar space is absent on both the right and left sides of TPL2, which is the more frequent condition when the mental foramen is anteriorly located (Table 4).

On the medial side of each ramus, the mandibular foramen is open with its aperture directed posterosuperiorly and there is a small, minimally-projecting lingula. On each side, the opening narrows to a shallow mylohyoid groove with no bridging. This open configuration is seen in the vast majority of western Eurasian and East Asian early modern humans (Table 4). The horizontal-oval form is found in only one East Asian (ZKD UC104) and one European (left side of Oase 1) modern human as well as in most recent human populations [36].

On the right ramus, both the coronoid process and the condyle are missing at the approximate level of the mandibular notch, making an estimate of its position unreliable. The left superior ramus is better preserved with a complete coronoid process but without a condyle. The lowest point of the mandibular notch lacks its posterior end, but its anterior and middle parts are in a position just posterior to the coronoid process; its lowest point is located anterior to the mandibular foramen. A symmetrical notch where the lowest point is approximately midway between the coronoid process and the mandibular condyle is consistent with the majority of early modern humans. In contrast, the lowest point of the notch is shifted posteriorly to a position just anterior to the neck of the condyle in the majority of archaic humans [37].

### Archaic human features: lateral corpus

In *norma lateralis*, the corpus is very robust, particularly with respect to its breadth at the position of the mental foramen (16.2 mm and 16.1 mm for right and left sides, respectively) and M_1_/M_2_ (18.3 mm and 18.6 mm for right and left sides, respectively). Across archaic and early modern humans, there is relatively little difference in corpus height at the mental foramen (p = 0.85) (Table 3). Corpus breadths, however, are significantly different between comparative samples (p<0.0001) (Table 3). At the position of the mental foramen and the M_1_/M_2_, a relatively broad corpus distinguishes archaic humans from the western Eurasian and eastern Asian early modern humans (Table 3). In these dimensions, TPL2 is most similar to the archaic humans, demonstrating a significantly broader corpus than the early modern human samples. At the position of the mental foramen, TPL2 has a corpus breadth (16.2 mm (right) and 16.1 mm (left)) that is broader than the average breadth of all Late Pleistocene archaic and early modern human samples. It falls within the range of variation of Late Pleistocene archaic humans (13.8–17.4 mm) and above the ranges of variation of East Asian and Western Eurasian early modern humans (11.4–12.8 mm and 11.1–14.1 mm, respectively) (S7 Fig). At the position of M_1_/M_2_, TPL2 (18.3 mm (right) and 18.6 mm (left)) falls above the range of variation for Late Pleistocene archaic humans (14.6–17.8 mm), East Asian early modern humans (11.7–14.1 mm) and western Eurasian early modern humans (12.4–16.0 mm) (Table 3).

### Geometric morphometric analysis

**Fig 3:**
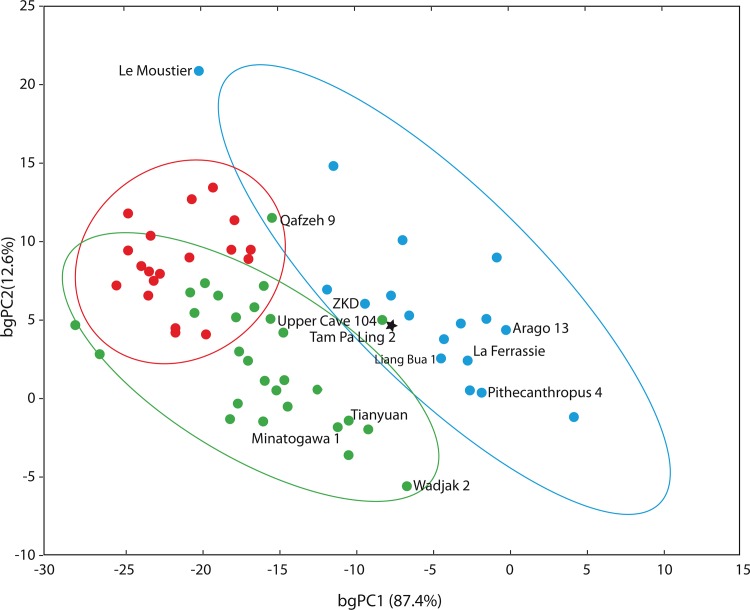
Between group principal component analysis (bgPCA). This plots the first two eigenvectors that separates three *a priori* defined groups: blue circles: Middle and Late Pleistocene archaic humans; green circles: Late Pleistocene early modern humans: red circles: Holocene humans. Fossils of particular interest are specified by name. Specimens included in the analysis are listed in S5 Table.

There is significant overlap of the 95% confidence ellipses (CE) for the early modern human and Holocene human groups, while the archaic human group is clearly separated in the morphospace represented by two non-zero eigenvectors (Fig 3). Only two early modern humans (Qafzeh 9 and Zhoukoudian UC 104) fall into the archaic human 95% CE. Tam Pa Ling 2 groups well within the archaic human CE, where it is also closely aligned with both the Zhoukoudian UC 104 and Zhoukoudian *Homo erectus* specimens.

Shape differences in bgPC1 are most strongly correlated with gnathion (the most inferior midline point on the mandibular symphysis); the superior transverse torus (the most posterior midline point on the superior transverse torus); mesial M1 projected onto the inferior border of the corpus and onto the mylohyoid line on the lingual aspect of the corpus; and the point on the alveolar bone at the M3. The archaic humans, with positive values on PC1, have relatively thick midline symphyses and pronounced mylohyoid lines that approach shelf-like configurations. In this way, TPL2 is more similar to the archaic humans, particularly in the expansion of the internal aspect of the mandibular body superior to the mylohyoid line. TPL2 also has a relatively thick superior transverse torus, despite being rejoined at the symphysis. Enlargement of the superior transverse torus has been related to overall mandibular size in some archaic humans and has also been noted as an archaic feature among Pleistocene *Homo*, whose symphyses were buttressed to withstand strong chewing and biting forces [38, 39].

## Discussion

The TPL1 cranium and TPL2 mandible were found in the same stratigraphic unit. Although direct dating of both specimens cannot confirm a strict contemporaneity, they belong to the same chronological frame of 63–44 ka. The TPL2 mandible demonstrates a mosaic of morphological affinities with respect to the comparative samples considered here. It has clear affinities with modern humans based on the presence of a chin with a protruding *tuber symphyseos* and lateral tubercles as well as other discrete traits of the mandible (Table 4). This derived morphology is juxtaposed, however, with archaic features such as the robusticity of the mandibular corpus—particularly at the M_1_-M_3_ level—and the relatively thick superior transverse torus, which more closely aligns TPL2 with the most robust archaic humans (Table 3 and S7 Fig). Geometric morphometric analysis also classifies TPL2 as an archaic human, due in large part to the morphology and robusticity of its corpus and maintenance of shelf-like tori combined with the shortness of the mandible.

Within the genus *Homo*, linear dimensions of the mandible—particularly those related to the lateral corpus—have been identified as taxonomically informative [40, 41]. In the western Old World, mandibular traits vary in frequency between Neandertals and early modern humans [35, 37, 42]. Lateral corpus breadth, however, consistently separates these groups with archaic humans having significantly larger breadths than early modern humans [43]. A lack of fossil material from Eastern Eurasia has prevented a thorough understanding of the variation of mandibular trait frequencies in archaic and early modern human samples more globally, but a recent analysis of the late archaic Xujiayao 14 and Penghu 1 mandibular remains from northern China and Taiwan (MIS 4–3 and MIS 6–3) has demonstrated differences between archaic and modern humans in the Eastern and Western Old World, at least in ramus morphology and anterior teeth [21, 52].

With the identification of new fossil remains and additional analyses of existing fossils, a mixture of archaic and modern morphology is increasingly frequent in the late archaic or earliest modern humans of all geographic regions. Penghu 1 shows mosaic traits (small condyles, low corpus and ramus heights vs. robust corpus and large anterior dental size with no chin) and affinities with Asian and non-Asian archaic mandibles [52]. While Xujiayao 14 primarily demonstrates ancestral, archaic traits, it has several uniquely derived features of modern humans [21]. Similarly, the Zhirendong mandible from southern China exhibits a modern human-like anterior symphysis and mental foramen position but maintains corporal robusticity similar to archaic humans [18]. In addition to Xujiayao 14, Zhirendong and TPL2 from Eastern Asia, significant morphological variation is present at the earliest anatomically modern human sites in Africa. While Omo-Kibish 1 (dated to ca. 195 ka) is completely anatomically modern, the Omo-Kibish 2 cranium demonstrates a mosaic of archaic and modern traits [44–46]. Similarly, both metric and non-metric traits of fossils from Herto, Ethiopia, dated to ca. 154–160 ka, are identified as intermediate between archaic and anatomically modern [47]. Similarly, Oase 1 and 2 from Romania, dated to ca. 35 ka, demonstrate key features found most commonly in archaic hominins despite being morphologically modern [48].

Genetic studies of Late Pleistocene hominins indicate increasingly complex population dynamics throughout the Old World, but particularly in Asia [49–51]. The mixture of archaic and derived morphology in the earliest modern humans has implications for population dynamics in the region. As with mosaic morphology in fossils from southern Africa [44–47], the early presence of this derived morphology at Zhiren Cave has been explained as the result of early modern human gene flow out of Africa and into East Asia with substantial admixture between dispersing populations and regional populations [18]. The morphological mosaics present in the Oase fossils have also been considered as possible evidence of mixed ancestry or as the result of processes of development and integration different from those seen in other archaic or modern *Homo* populations [48]. As these examples continue across the Old World, the morphological differences identified in mandibular morphology between Pleistocene archaic and modern humans may be a useful typological distinction, but the biological meaning underlying this dichotomous comparison is increasingly problematic [48]. Our construction of these categorical divisions may indicate population-level processes in some cases; however, it is likely that the presence of “mosaic” morphology conflates anatomical variation and biological processes resulting in the emergence of modern humans.

Fossil evidence at Tam Pa Ling supports an early dispersal out of Africa and into Southeast Asia by the middle Late Pleistocene [1]. The mixture of features identified in the TPL2 mandible are associated with both archaic and modern human comparative samples. While these fossils may be further evidence of the overlap in traits and varying trait frequencies between archaic and early modern humans [35, 37, 42], archaic traits of TPL2 and the fully modern human morphology of TPL1 (with no archaic features) more likely suggest that a large range of morphological variation was present in early modern human populations residing in the region by MIS 3. They also provide evidence for continued questioning of the validity of a typological paradigm that pits archaic against modern anatomy given the growing number of Late Pleistocene fossils that present morphology consistent with both comparative samples.

## Materials and Methods

The TPL1 and TPL2 fossils are housed at the Heritage Department of the Ministry of Information, Culture and Tourism of Laos in Vientiane. All necessary permits for excavation were obtained from the Heritage Department, and excavation and research complied with all relevant regulations of the Ministry of Information, Culture and Tourism. A brief description of the preservation and morphology of the TPL2 mandible is provided below, with additional information included in S1 Text. A comparative assessment of TPL2 was performed using discrete traits, linear and angular morphometrics following [23] and geometric morphometrics [24, 25] (Fig 3 and S7 Fig; Tables 3, 4, and S3 Table). Due to the mixed archaic and modern human features of TPL2 and its geologic age, comparative samples included archaic and early modern humans from the Middle and Late Pleistocene, with a particular emphasis on those available from East and Southeast Asia (see below and S5 Table).

Late Pleistocene samples were evaluated for differences in linear dimensions and discrete traits using model II analysis of variance (ANOVA) with post-hoc Bonferroni tests for multiple comparisons. Comparative samples included the following: 1) Late Pleistocene archaic humans (i.e. Neandertals); 2) Late Pleistocene early modern humans (EMH) from Western Eurasia and Africa; 3) Late Pleistocene EMH from East Asia.

The geometric morphometric analysis was performed using 67 hemi-mandibles. Data were collected in the form of 51 3-D coordinates representing 17 landmarks (S8 Fig and S4 Table). Cross-sectional imaging of TPL2 was generated using the microfocus tube of the micro-CT scanner “v|tome|x L 240” (GE Sensing & Inspection Technologies Phoenix X|ray) and the AST-RX platform (*Accès Scientifique à la Tomographie à Rayons X*, *MNHN*, *Paris*). The complete TPL2 specimen was numerized at a resolution of 60 μm.

Sample composition for this analysis was expanded to include both Middle Pleistocene and Holocene data, but was limited to specimens for which comparable 3-D data were available (S5 Table). Comparative samples included 1) Middle and Late Pleistocene archaic humans; 2) Late Pleistocene EMH from all geographic regions; and 3) Holocene humans from China.

Between-group principal component analysis (bgPCA), the projection of data onto the principal components of the group means [26], was used to identify axes in shape space that best discriminate between the three *a priori* defined comparative groups described above (performed using R free software; see http://www.r-project.org). The samples were separated by computing a covariance matrix of the group means and projecting all specimens into the space spanned by the first two eigenvectors of this matrix. The advantage of this method relative to canonical variates analysis is that the axes remain orthogonal and data do not have to be of full rank. Tam Pa Ling 2 was not included in the calculations of the PCs, and this fossil was subsequently projected onto the PCs to identify its closest neighbors. This step is critical to the statistical analysis because its removal from the database enables one to build a model that is free of the parameter to be judged and to avoid bias in assigning group membership.

## Supporting Information

S1 Text Supporting Text. (DOCX)

S1 Video Animation of the TPL2 mandible. This video shows the teeth segment extracted.
(AVI)

S1 Fig View of south entrance to TPL with excavation area in the lower part of the background. Photo taken from the south, looking towards the north.
(TIF)

S2 Fig Views inside TPL cave. Top: Photo of the main gallery of TPL from the cave entrance, looking north. Test pits are shown on the left (trenches 1 and 2); excavation site is shown on the right (trench 3). Bottom: Photo of the main gallery of TPL from the west looking east. Test pits are shown in the foreground (trenches 1 and 2); excavation site is shown in the background (trench 3).
(TIF)

S3 Fig Human fossil remains designated as TPL 1. Elements: (A) frontal bone in *norma facialis*; (B) in *norma verticalis*; (C) in *norma basilaris*; (D) occipital bone in *norma verticalis*; (E) occipital bone in *norma basilaris*; (F) right parietal bone in *norma verticalis*; (G) right parietal bone in *norma basilaris*; (H) left temporal bone with partial mastoid in *norma lateralis*, external; (I) left temporal bone with partial mastoid in *norma lateralis*, internal; (J) maxillae in *norma facialis*; (K) maxillae in *norma verticalis*; (L) maxillae in *norma basilaris*.
(TIF)

S4 Fig Photo of the TPL2 mandible taken in situ upon discovery. (TIF)

S5 Fig Photo of the TPL2 mandible found in two halves in situ. (TIF)

S6 Fig 3D reconstruction of TPL2 mandible. (a) the complete mandible; (b) the complete teeth segment extracted from the mandible corpus; (c) the complete teeth segment extracted in anteroposterior view.
(TIF)

S7 Fig Measure of corpus height versus corpus breadth at the mental foramen (in mm). Red circles: Late Pleistocene archaic humans; green circles: Middle Paleolithic EMH; purple circles: western Eurasian EMH; orange circles: East Asian early modern humans; yellow star: TPL2. Black line is the linear regression line.
(TIF)

S8 Fig Landmarks position. Drawing showing the position of the landmarks used for the geometric morphometrics analysis.
(TIF)

S9 Fig Plan of the TPL excavation (bottom right). Stratigraphic sections from trenches 1–3 are shown and correlated.
(TIF)

S10 Fig Line drawing of relationship between excavation site (trench 3) and test pits (trenches 1 and 2). (TIF)

S11 Fig The results of OSL single-grain analysis for samples TPL4-8-OSL. (a) representative shine down plot and (b) dose response curve is presented for sample TPL4-OSL. (c-g) single-grain distributions presented as radial plots for samples TPL4-OSL (c), TPL5-OSL (d), TPL6-OSL (e), TPL7-OSL (f), TPL8-OSL (g).The shaded area represents palaeodose values within 2Ïƒ of the central value for each distribution, while the solid line represents the palaeodose value determined using the minimum age model (MAM). (h) The relative profile likelihood for the MAM processed for sample TPL4-OSL displaying the optimization of the model and the generated palaeodose of 86 ± 5 ka.
(TIF)

S1 Table TPL2 linear and angular dimensions. Measurements are in millimeters unless otherwise indicated. Estimated values are in parentheses.
(DOCX)

S2 Table Discrete observations of the TPL2 mandibular corpus. (DOCX)

S3 Table Discrete traits of the TPL2 mandible and their frequency in comparative samples. Comparative samples include Late Pleistocene (Late Pl.) archaic humans, Middle Paleolithic early modern humans (EMH) and Late Pleistocene early modern humans from East Asia (Late Pl. East Asian EMH) and western Eurasia (Late Pl. Western Eurasian EMH).
(DOCX)

S4 Table Landmarks taken on hemi-mandibles for geometric morphometric analysis. (DOCX)

S5 Table Fossils included in comparative analyses. Middle Pleistocene archaic and Holocene samples used only for geometric morphometric analysis.
(DOCX)

S6 Table OSL single-grain rejections. (DOCX)

S7 Table 14C age estimates. (DOCX)

S8 Table Mandibular molar crown diameters for TPL2 and comparative samples. Summary statistics provided as mean ± standard deviation, N. BL = buccolingual crown diameter; MD = mesiodistal crown diameter. Comparative samples as in S3 Table.
(DOCX)

S9 Table Rodent faunal list for Tam Pa Ling. On the 366 remains, 249 have been identified as shown in the table (NISP). The 27 remains of undetermined Murinae could be grouped as small, medium and large size murin, with the calculated MNI for each size.
(DOCX)
