# Supplementary material for: Early Modern Humans and Morphological Variation in Southeast Asia: Fossil Evidence from Tam Pa Ling, Laos
Source: PLoS One. 2015 Apr 7;10(4):e0121193. doi: 10.1371/journal.pone.0121193 (PMC4388508; doi:10.1371/journal.pone.0121193)
Supplement: S1 Text — (DOCX) [file pone.0121193.s001.docx]

Supporting Information for

Early modern humans and morphological variation in Southeast Asia: fossil evidence from Tam Pa Ling, Laos

1. Supplementary Text

Excavation history and site summary

The lower part of the south-southeast facing cliff of Pa Hang Mountain was extensively excavated in 1934 by Jacques Fromaget, Chief of the Geological Service of Indochina. At this time, the Tam Hang rock shelter was identified, and archaeological and paleontological excavations were undertaken. Middle Pleistocene mammalian fauna were excavated from breccia deposited in karstic networks exposed along the 100-m length of the Tam Hang rock shelter ([1-5](#_ENREF_1)). Archaeological artifacts and human remains dated to ca. 13.5 ka were recovered from recent sediments deposited under the shelter ([6-10](#_ENREF_6)).

Based on Fromaget’s publications, the location of Tam Hang was re-identified by Thongsa Sayavongkhamdy of the Department of National Heritage, Lao P.D.R. and reopened for study in 2003 by two of the authors (F. Demeter and A.-M. Bacon). The site has been excavated by several of the authors annually since 2007 (A.-M. Bacon, F. Demeter, P. Duringer, T. Sayavongkhamdy, L. Shackelford, E. Patole-Edoumba, J.-L. Ponche, K. Westaway). While surveying the upper part of the Pa Hang Mountain in November 2008, P. Duringer discovered Tam Pa Ling (TPL) cave. Charcoals from three test pits in TPL were collected and radiocarbon dated at the Illinois Geological Survey in January 2009 (see below). Based on AMS radiocarbon dates, trench 3 was opened as the primary excavation site during the next field season (December 2009), and field work at the site has continued annually. The partial skull of an early modern human (TPL1) was recovered in December 2009, and details of this fossil have since been published ([11](#_ENREF_11)) (Fig. S3). The TPL2 mandible was unearthed during the 2010 field season (Fig. S4, S5). Sediments, charcoals and calcite were collected each season for dating (OSL, U/Th-series and ^14^C) in order to increasingly refine the chronology of the site.

Stratigraphy and sedimentology

The Tam Pa Ling cave is located at the highest part of the Pa Hang Mountain (elev. 1170 m), approximately 80 m from the Tam Hang cliff and rock shelter. The south facing entrance is hidden behind dense vegetation, and less than 20 m of limestone cover the cave. Tam Pa Ling has a single, main gallery that lies at the base of a steep, 65-m-long slope (Fig. 1, S1). The 30 m-length of the gallery is oriented in a north-south direction. The 40 m-width of the gallery is oriented in a roughly east-west direction and divided into two chambers (East and West chambers) by a large, stalagmite column (Fig. S2). Three trenches were excavated on the gallery floor to examine the stratigraphy and sedimentology of the cave: trenches 1 and 2 at the west end of the cave and trench 3, the primary excavation, at the east end of the cave (Fig. S2, S9, S10). The stratigraphic markers from the primary excavation site (trench 3) can be followed laterally east and west for at least 8 meters without disruption of the sediment layers. The stratigraphy is further continuous with an additional test pit excavated in the far eastern part of the cave in 2012.

The sedimentary deposits inside the main chamber of the TPL cave are predominantly soft, wet and rich in brown clay. Detailed analysis of the three trenches identified three main types of deposits: sandy clays, silty clays, and several white calcitic argillaceous layers. Iron/manganese concretions and pisolites are frequent through the stratigraphic section (Fig. 1). From the top of the section to a depth of ∼2 m, brown sandy clays alternate with thin layers of silty clays. The lowest part of the section (from ∼2 m to bottom) is formed by continuous, homogenous, silty clay deposits (Fig. 1). Granular clasts to large-sized limestone blocks (up to 2 m along the main axis) are scattered vertically throughout the trench, with the number of large blocks increasing with increasing depth. The primary source of these limestone blocks is the collapsed roof. Together with such scattered clasts, the clay-dominated cave infillings are punctuated by six white, silty, clayey levels (Fig.1), which can be followed without discontinuity across the excavation to the eastern cave wall (over 10 meters). These levels accumulate a clayey, powdery calcite, such as pseudo-hard grounds or ‘moonmilk,’ recording possible in situ calcite precipitation. Among them an almost pure, yet powdery calcitic layer underlines the transition at ∼2-1.8 m between the lowermost and the uppermost parts of the section.

The bulk deposits (20-cm sampling interval) are made of variable amounts of quartz, calcite and clay minerals together with iron oxy-hydroxides, goethite, small amounts of hematite and traces of gibbsite. Quartz is derived from the surrounding Devonian arkosic sandstone. Calcite is primarily derived from the cave limestone, as well as from shell fragments of terrestrial gastropods and small-sized vertebrates such as rodents and amphibians. Their red color is due to oxides coatings.

The mineralogical assemblage of the clay fraction of deposits (<2 µm) does not vary drastically throughout the section (Fig.1) and is predominantly made of vermiculite and kaolinite. Illite, fine quartz and iron oxides also occur in low frequency. Vermiculite comprises hydroxyl-Al interlayers (persistence of water molecules and cations). Like the silty clays, which become more clay-rich above the calcitic intervals, the relative vermiculite content in the clay also increases as you move upward, at the expense of kaolinite and illite. The clearer rise from 1.4 m to the top of the section could reflect increasing hydrolyzing climatic conditions.

Vermiculite and kaolinite are widespread clay minerals in soils and, when associated with iron and aluminum oxides, indicate hydrolyzing leaching conditions under humid sub-tropical climate. These minerals are characteristic of "ferralsols-nitisols" strongly weathered soils. Furthermore, vermiculite and kaolinite could indicate the mixing of parent sources for the soil components that formed the cave deposit. Vermiculite is more often the result of weathering of biotite or illite-bearing rocks, such as the surrounding arkose and sandstone found close to the studied outcrop or in Pleistocene karst breccias. Kaolinite is a common weathering by-product of a wide variety of parent rocks, including limestone. Due to the small number of sinkholes present in this cave environment and the stratigraphy and morphology of the cave, it is assumed that the source area for these sediments is the front (south-facing) entrance of the cave. Cave deposits most likely originated from the muddy mound at this entrance.

The sediments represent periodic and localized slopewash deposition that has formed distinct and regular layers throughout the sediment column. There is no evidence of rapid deposition; instead the layers alternate between fine and slightly coarser fractions that could reflect -wet and dry season influxes of sediment. The sedimentation rate is difficult to quantify because deposition is obviously intermittent. There is no evidence of bioturbation, post depositional mixing, slumping or reworking, and the preservation of distinct layers suggests minimal disturbance since burial. The sedimentary bedding of all three pits is rigorously parallel to the dip of the soil of the cave, indicating that despite being deposited by slopewash after burial, the sediments and associated fossils have been undisturbed (Fig. S9, S10). The white, calcite-rich, continuous layers represent ‘moonmilk’ deposits that may be linked to the temporary cessation of cave infilling in response to hiatus that could be driven by climatic or/and hydrodynamic evolution.

Strategy for developing the TPL chronology

The original chronology for the TPL stratigraphy was focused on the same level as the uppermost skeletal evidence, the skull and mandible (TPL1 at 2.35 m and TPL2 at 2.65 m), which bracket the age range of sedimentary deposition between 48-46 ka, with the cranium laid down by at least 46 ka. This chronology was established using luminescence dating (both optically-stimulated luminescence (OSL) and red thermoluminescence (TL)). In addition there are some radiocarbon results on charcoal, that have been interpreted as old carbon, that has been washed into the cave environment (i.e., not burnt in-situ) and therefore represent maximum ages for sediment deposition, however as the lowermost two results are on the cusp of the radiocarbon barrier the ‘true’ maximum age of the material could actually be much older for these samples. Therefore the ^14^C results are maximum ages in the context of sedimentary deposition in the cave but the lowermost two samples are effectively minimum ages in the respect of the limitations of the technique and have been presented as >40 ka. Despite this, the ^14^C results will be discussed from now on as maximum ages. Furthermore, a minimum U/Th-series age on bone ([11](#_ENREF_11)) provides further supporting evidence for the antiquity of the fossil evidence (see later section for this discussion). Despite the stratigraphic consistency and some agreement between techniques, the chronology has been contested (([12](#_ENREF_12)) with the reply in ([9](#_ENREF_9))) with questions raised over the apparent inverse chronology and a large period of sedimentary hiatus in the stratigraphy in the order of ~44 ka. Every effort was made to check the accuracy of this chronology using independent age estimates from different material but this proved unsuccessful; the capping ‘moonmilk’ layer was far too reprecipitated to obtain a reliable U/Th-series result and due to the structure of the cave and its role as a ‘sink’, carbon in the sediments would not have been derived from *in situ* burning and therefore only provides a maximum age for the deposition of each layer (but this maximum age is capped by the constraints of the technique for the deepest samples). Therefore, it was decided that the OSL chronology would be extended upward through the section with the aim of extending the chronology, addressing the apparent inverse chronology, confirming the integrity of the deposits, quantifying the rate of sedimentation and investigating the apparent hiatus period that lies between the upper OSL age of 46 ka and the radiocarbon result of ~2 ka. In this respect, sampling was concentrated on either side or middle of the apparent hiatus period. Five additional OSL samples were collected at depths of 2.0 m (TPL4-OSL), 1.80 m (TPL5-OSL), 1.50 m (TPL6-OSL), 1.20 m (TPL7-OSL) and 0.60 m (TPL8-OSL) .Furthermore, four additional charcoal samples were collected for radiocarbon dating to establish maximum ages for sediment deposition at depths of 1.47 m, 1.75 m, 1.80 m and 1.97 m, and a sample was taken at the tip of an overhanging buried stalactite (TPL-F3) at 2.30 m, which corresponds with the depth of the cranium and provides a maximum age for sedimentary infilling at that depth.

Luminescence dating is the most appropriate and useful sediment-based dating technique for establishing the time since the associated minerals were last exposed to sunlight. Optically-stimulated luminescence techniques measure the time at which sediments were either last heated (e.g., burnt or fired) ([13](#_ENREF_13)) or exposed to sunlight during transport or deposition (e.g., by wind or water) ([14](#_ENREF_14)) via the stimulation of minerals using heat or light. This is a trapped charge technique whereby a light sensitive signal in the form of an electron is “trapped” in defects in the crystal lattice of minerals such as quartz and feldspar. This energy builds up slowly in low dose rate sediments (e.g., 1 Gy/ka) and is stored in the crystal during a period of burial in the sediment column. The amount of trapped energy is proportional to the amount of naturally occurring ionizing radiation found in the surrounding sediment and the amount of exposure time during the burial period ([14](#_ENREF_14)). As the signal is reset to zero by sunlight – it represents a clock to measure the time since last exposure ([15](#_ENREF_15)). This makes these techniques unique amongst dating methodologies as they can be used to estimate the time elapsed since sediments were buried ([15](#_ENREF_15)), and as most processes or events are based on the deposition of sediment, depositional ages represent an extremely desired commodity in palaeoanthropological research.

Luminescence dating methods

1. Sedimentary context

The stratigraphic integrity of the site has been maintained despite periodic slopewash entering the cave. The sediments contain no evidence of cut and fill processes, channels being eroded or large erosion contacts, suggesting that the water from slopewash processes remains on the surface and does not interfere with the underlying sedimentary layers ([11](#_ENREF_11)). As such, the radiocarbon age estimates provide a maximum age for fossil deposition (apart from the two lowermost samples that are capped by the limitations of the technique and are presented as >40 ka), whilst the OSL single-grain age estimates should provide a result close to the ‘true’ burial age.

2. Sample collection and preparation

Sediment samples for luminescence dating (laboratory code TPL4-6) were collected from a cleaned exposure of the TPL excavation using either opaque plastic tubes that were hammered horizontally into the sedimentary section and then covered in light-safe wrappings (TPL4-6-OSL) or as bulk samples that were collected in subdued red light conditions (TPL7-8-OSL). Additional samples of sediment were collected from within 30 cm of the OSL sample and subsequently combined for water content and environmental radioactivity determinations. Quartz grains of 180-212 µm in diameter were separated from the matrix under dim red illumination using standard purification procedures, including a final etch in 40% hydrofluoric acid for 45 min to remove the external alpha-dosed rinds ([14](#_ENREF_14)). The acid-etched 180-212 µm grains were loaded on aluminum discs for single-grain OSL analysis, with a total of 100 grains per aliquot. A total of 8-10 aliquots were processed for each sample with 800-1000 grains.

3. Single-grain OSL dating

Previous work on this site has demonstrated that the sedimentary characteristics are suitable for OSL techniques, and there is an internal consistency between different wavelengths of the luminescence signal producing agreement between OSL and red TL ages. In addition, the SAR procedures including a preheat of 260°C are appropriate as demonstrated by the successful dose recovery measurements. The relatively high overdispersion values of the single-aliquot results suggested that single-grain analysis is required ([11](#_ENREF_11)). Therefore, for the current analysis only single-grain techniques were employed using the same measurement conditions.

The TL-DA-20 Risø unit containing the single-grain attachment was utilized for all OSL single-grain analyses. The attachment was used in combination with custom-made aluminum discs containing 100 precision-drilled 300 μm (depth and diameter) holes in a ten-by-ten grid ([16](#_ENREF_16), [17](#_ENREF_17)). A calibrated ^90^Sr/^90^Y source was used to perform laboratory irradiations at a dose rate of between 10.45-10.28 Gy/min (run in two separate batches) for the aluminum single-grain discs. Ultraviolet emissions were detected by an Electron Tubes Ltd 9235QA photomultiplier tube fitted with 7.5 mm of Hoya U-340 filter. The composition of these discs compared with the stainless-steel discs warranted a dose correction to account for differences in dose absorption due to backscattering and differences in the distance of the grains to the beta source ([16](#_ENREF_16), [17](#_ENREF_17)). Therefore, detailed single-grain analysis of the aluminum disc/beta source combination was calibrated using calibration quartz (4.81 Gy batch 60 from Risø) revealing slight variations in the spatial homogeneity of the source ranging from 8.7-12.8 Gy/min, so grain corrections were applied to the generated D_e_ values. Each of the 180-212 µm-sized quartz grains was stimulated for 2 s using a 10 mW 532 nm Nd:YV0_4_ solid-state diode-pumped green laser, which was focused to deliver 50 W/cm^2^ of power to a 20 μm-diameter spot, and the ultraviolet emissions were detected by an Electron Tubes Ltd 9235QA photomultiplier tube fitted with 7.5 mm of Hoya U-340 filter. The most light-sensitive component of the OSL signal was obtained from the initial 0.12-0.3 s of stimulation (6-15 channels), and the background count rate was estimated from the last 1.62-1.9 s (81-95 channels).

The single-aliquot regenerative dose protocol (SAR) ([18](#_ENREF_18)) was employed for all OSL measurements. This revolutionary technique uses paired measurements (a natural/regenerative and a test dose measurement) to account for the sensitivity changes induced by the regenerative cycles of bleaching, dosing and heating. In total, the OSL paleodoses of 5800 individual grains were measured using a modified single-aliquot regenerative-dose protocol described in detail elsewhere ([19](#_ENREF_19), [20](#_ENREF_20)). The same preheat conditions as described for previous analysis were employed including a IR diode wash prior to OSL measurement along with a hot optical wash (280°C blue diodes stimulations for 100 s ([21](#_ENREF_21)) and as tested by the dose recovery experiments ([11](#_ENREF_11))) and two extra regenerative cycles (OSL-IR depletion ratio test) to assess the IR component of each grain ([22](#_ENREF_22)). The single-grain data have been presented as radial plots in Fig. S11. The most robust tests of the procedures employed for the SAR measurements is the dose recovery test for internal consistency and preheat plateau test to determine optimum preheat conditions.

4. Recovering a known dose

Previous dose recovery tests were conducted using single-aliquots^4^. Similar dose recovery tests were performed on 300 single-grains of quartz to test the procedures employed. After initial bleaching at low temperatures (100 s of blue diodes at 50°C) a dose of 100 Gy was administered as the surrogate natural and the same SAR protocol was employed as described for the single-grain analysis. Out of the 300 grains measured the surrogate natural was recovered to within 10-15% for the 20-40 accepted grains with an average of 99 Gy and an overdispersion of 4%. All data and plots for this test were presented in ([11](#_ENREF_11)).

5. Single-grain rejection criteria

Single-grains were accepted and rejected according to the criteria of ([23](#_ENREF_23)). Grains were rejected if the test dose error exceeded 10% and if the signal was smaller than 3σ above the background value. Grains were also rejected if the associated recycling ratios were greater than 10% either side of unity or if the OSL-IR depletion ratio was more than 2σ less than unity. This provided a range of grains that could then be analyzed individually using Analyst_TM_ program. Further rejections were implemented for supersaturating grains ([24](#_ENREF_24)), grains that displayed recuperation exceeding 5% of the natural signal, excessive increasing or decreasing sensitivity changes in the test dose responses in relation to the natural, the presence of a desensitizing slow component that displays a difference between the natural and first regenerative decay curve, and the dominance of a medium component in the shape of the natural shine down (Fig. S11 and Table S6).

6. Modern day analogue

A modern sample (TPL-MOD) was collected from directly outside the cave entrance in a location, which afforded partial exposure to sunlight. This area is thought to be the source for the sediments inside the cave and therefore provided a useful location to test the assumption of luminescence dating that most of the grains being brought into the cave were bleached prior to deposition. Once processed using the same methods as described above, the sample was analyzed using 12 small aliquots. The OSL emissions returned a paleodoses in the range of 0.2-1.5 Gy suggesting that the majority of the sediment is being bleached before entering the cave; however, we cannot assume that this has always been the case over the last ~40 ka. If slopewash deposition occurred at night during a storm there would be less opportunity for bleaching –and a strong likelihood that some of the grains have only been partially bleached – hence the large proportion of grains with large residual doses (100’s Gy). These results indicate that the use of a minimum age model (MAM) would be the most appropriate for D_e_ determination, but the modern day analogue indicates that at least some of the grains have been bleached prior to deposition so that the paleodose estimation derived from the MAM are valid.

7. Estimation of the paleodose using statistical models

The single-grain distribution indicates that the sediment contains a mixture of higher and lower dosed grains ranging from 266-37 Gy (TPL4), 234-40 Gy (TPL5),172-10 Gy (TPL6), 9-70 Gy (TPL7), 5-45 Gy (TPL8) very similar to those observed in TPL1-3 ([11](#_ENREF_11)). This distribution could be interpreted as due to a mixing of grains from different layers, small scale variations in the beta dose rate experienced by different grains or a difference in the amount of bleaching time prior to deposition. The opportunity for beta heterogeneity is small due to the homogenous composition of each layer, but the opportunity for incomplete bleaching of each grain is high due to the deposition occurring via slopewash processes into the cave environment. It is not unexpected to find partially bleached grains in slopewash, fluvial or colluvial deposits ([25](#_ENREF_25)), however, the fully bleached grains represent the true burial age of the skeletal material. The central age model (CAM) ([26](#_ENREF_26)) is only suitable for samples that are well bleached, generally with an overdispersion of less than ~20% and is consistent with single-component D_e_ distributions ([19](#_ENREF_19)). These samples have fairly high overdispersion values of 33-40%. The modern analogue sample from outside the cave entrance returned an age close to zero thus there is good potential for the samples to bleach, but there is still a strong likelihood that the sediments were deposited rapidly without being bleached in the cave entrance, e.g., during storm events, or rapid deposition at night. Therefore, based on the distribution of single-grain paleodoses and our understanding of the depositional environment we have adopted the most conservative approach by employing the MAM ([27](#_ENREF_27)). This model was implemented using an inherent overdispersion of 10% added to the error of each individual paleodose estimate, and the relative profile likelihoods are plotted as insets on the radial plots. This model is sensitive to the presence of low dose grains so the grains that received the most complete bleach will influence the final D_e_ and therefore provide a depositional age that most closely represents the timing of fossil deposition in the cave.

8. The equivalent dose distributions

The samples are not as scattered as would be expected from a typical slopewash or fluvial sediment with a relatively small range of D_e_ values. This could indicate that only a small degree of partial bleaching has occurred i.e., there is not much difference between the fully bleached and partially bleached grains, and that prior to this depositional event a large proportion of the grains must have undergone a substantial bleaching event. However, this does not agree with the context or our sedimentological interpretation of the site, therefore in the interests of using the most parsimonious interpretation the MAM is still valid. The resulting paleodoses are all stratigraphically consistent and display a clear age-depth relationship.

9. Dose rate

Concentrations of ^238^U, ^235^U, ^232^Th (and their decay products) and ^40^K were measured by Geiger Muller beta multi counting of dried and powdered sediment samples in the laboratory and a portable gamma spectrometer in the field. It is assumed that the dose rate measured at the time of sample collection has prevailed throughout the burial period. The corresponding (dry) beta and gamma dose rates were obtained using the conversion factors of ([28](#_ENREF_28)) and the beta-dose attenuation factors of ([29](#_ENREF_29)), and an effective internal alpha dose rate of 0.03 Gy ka^-1^ was assumed for each sample and included in the total dose rate ([30](#_ENREF_30), [31](#_ENREF_31)). Cosmic-ray dose rates were estimated from published relationships ([32](#_ENREF_32)), making allowance for the density and 50 m thickness of the limestone roof above the cave deposit, the geometry of the limestone shielding, the sediment overburden at the sample locality (~2.0-1.50 m), the altitude (~1170 m above sea level) and geomagnetic latitude and longitude (22° and 102°) of the sampling site. The original TPL samples were estimated using high resolution gamma spectrometry and there is good agreement between these and the new beta and gamma counting system.

10. Disequilibrium in the cave environment

High resolution gamma spectrometry of the TPL cave sediments previously indicated that there is some disequilibrium in this cave environment (see ([11](#_ENREF_11)) for a detailed discussion). Therefore to accommodate this additional source of uncertainty the error margin on the dose rate has been increased by 6% for each sample.

11. Estimations of water content

Material collected around the sampling areas was used for laboratory estimations of the present-day water content. The material was placed in a (pre-weighed) beaker and weighed, to determine the wet sample weight. The sample was then dried in an oven at 40°C for several days and reweighed to determine the dry sample weight. Deducting the weight of the dry sediment and the empty beaker from the original wet sample weight produced a value for the total weight of water loss during drying. The *in situ* water content was then expressed as the weight of water divided by the weight of the dry sediment. These measurements contributed to the assessment of water content variability during the period of burial. In addition, field observations and assessments of past variability were conducted to assist in the final estimation of the annual dose. The total dose rate was calculated using long-term water contents of between 20 ± 5 % and 30 ± 5 %, which is close to the measured (field) water content of between 28-47 %. These are similar to the previous estimations relative to the depth in the stratigraphic column. Water content variability during the period of burial was assessed according to location, sediment depth and sediment composition. During present-day conditions, northern Laos usually has 6 wet months and 6 dry months, and is ‘wet’ roughly 55% of the year; Luang Prabang receives about 1,360 mm of rainfall annually. The depths of the samples in the cave environment also influence their water contents, being either covered (e.g., superficial cave sediments that are dry and cracked in the dry season, and saturated in the wet season) or deeply covered (e.g., deeply buried cave sediments that are constantly saturated all year round, but were once on the cave floor surface), with composition also playing a role. This variability was considered when estimating the errors associated with the water content for each sample to ensure that the errors covered the range of likely values. As a starting point superficial cave sediments would be expected to be ~20 ± 5 %; and basal cave sediments ~40 ± 5 % of water content. These values were later modified after including the laboratory estimations of present day water content. The water content has also been conservatively estimated to accommodate the potential range of water content values of between 20-30%. We attribute these high values to the steep entrance slope and the continuous slopewash processes that channel the surface run-off from the limestone tower down into the cave. This water tends to accumulate in the finer layers as the clays and silts prevent the water from seeping down through the layers.

The OSL ages were calculated by dividing the OSL paleodose derived by the MAM by the corresponding total dose rate (Table 1 in main text).

^14^C methods

The standard procedure ABA (acid-base-acid) pretreatment was used for AMS ^14^C dating of TPL charcoal samples. The same pretreatment was also applied to the ISGS ^14^C-free wood background and wood working standards that include IAEA C5 (Two Creek forest wood), FIRI- D (Fifth International Radiocarbon Inter-comparison D wood), and ISGS Reily AC (about 3 half-life wood) samples.

All samples were boiled for 1 hour in 2M HCl and rinsed to neutrality using DI-water; then socked in 0.125 M NaOH for an hour and rinsed to neutrality using DI-water; then socked in 2M HCl for 30 minutes and rinsed to pH 6 using DI-water. Samples were dried in oven overnight at 70°C. About 2-3 mg materials of each unknown samples and wood background and working standard samples were placed into preheated quartz tubes for sealed quartz tube combustion at 800°C with minimal amount of CuO granules (~0.5 g). Quartz tubes were preheated at 800°C for 2 hours, and CuO granules were preheated at 800°C one day before usage. The combustion was programmed for 2 hours at 800°C. Then samples were cooled from 800°C to 600°C for 6 hours to allow Cu to reduce the NxO to nitrogen gas. The purified CO_2_ was then collected cryogenically under less than 5 mTorr vacuum condition for AMS ^14^C analysis. Purified CO_2_ was submitted to the Keck Carbon Cycle AMS Laboratory of the University of California-Irvine for AMS ^14^C analysis using hydrogen-iron reduction method ([33](#_ENREF_33)). A split of purified CO_2_ was also analyzed for δ^13^C values using the in-house Finnegan 252 IRMS (isotope ratio mass spectrometer) with a dual inlet device. All results have been corrected for isotopic fractionation according to the conventions of ([34](#_ENREF_34)), with δ^13^C values measured on prepared graphite using the AMS spectrometer. Sample preparation backgrounds (at least 2 aliquots) have been subtracted, based on the measurements of ^14^C-free wood blank. The AMS analysis indicated that after background subtraction, wood working standards of IAEA C5, FIRI-D, and Reiley AC yielded target values with 1σ deviations. The ^14^C dates are calibrated using the IntCal13 ([35](#_ENREF_35)).

U/Th methods on bone

A bone fragment from the ascending left ramus of the mandible TPL2 and more specifically from the part forming the left condyle, which was broken into pieces, was sampled for U/Th-series dating (LS12-B1-B5 - measured at the U-series Laboratory at the University of Queensland, Australia) to confirm the age of the fossil itself. High-precision solution Element (e.g. U, P, Sr, REE)/Ca ratio analyses was conducted using quadropole Inductively coupled plasma mass spectrometry (ICPMS). U/Th-series measurements were made on micro-drilled sub-samples, using analytical procedures described elsewhere ([36](#_ENREF_36), [37](#_ENREF_37)). This was followed up by using representative samples to do solution U/Th dating. The ^230^Th/^234^U ages were calculated using half-lives of 75,380 years (^230^Th) and 244,600 years (^234^U). The U/Th-series dating of the bone fragment proved challenging as it could not be microdrilled for U/Th-series profiling due to its porous nature. As such, the analysis was conducted on small handpicked fragments that contain inseparable post-fossil secondary calcite overgrowths, and the bone piece is porous (unlike tooth dentine/enamel which are solid), so the individual fragment ages should represent minimum ages for the fossil itself. The five fragments give ages ranging from 36.2 to 43.7 ka. This provides a minimum age of >35 ka but it is expected that porous bones tend to give apparent U/Th dates that are significantly younger than the true age of the fossil i.e., the fossils cannot be any younger than 36-44 ka, and should in fact be older when considering the depositional ages and previous U/Th dating.

The U/Th dating of the frontal bone for TPL1 could also not be microdrilled for U/Th profiling and despite being a maximum age (or the upper age range) for sediment deposition in combination with the OSL results ([11](#_ENREF_11)), it has been presented as a minimum age for bone formation due to the possibility that the bone represents an open system with Uranium migrating into the bone after deposition. Due to the inability to conduct U/Th profiling on both of these samples to test for these effects – we do not hold much confidence in these results and have subsequently been presented as supporting data for the main OSL chronology.

TPL2: Descriptive analysis

1. Preservation.

The corpus has significant damage to the alveolar bone immediately surrounding the break at the midline symphysis (see below), but otherwise shows only minor post-mortem scratching and abrasion. The right ramus is broken at the level of the sigmoid notch and is missing the mandibular condyle and coronoid process. The left mandibular condyle is broken at the level of the sigmoid notch although the coronoid process is complete and the anterior portion of the sigmoid notch is present. A remaining portion of the condyle was used for U/Th-series dating (see Context and dating).

The superior corpus and alveolar margin are broken at the midline, leaving a gap in the area of the central incisors with a maximum width of 7.6 mm and maximum height of 12.5 mm. The alveolar bone of the central incisor tooth sockets is absent and these teeth have been lost post-mortem. The sockets for both lateral incisors are intact (their presence has been verified on CT scan, Fig. S6). On the right side, the alveolar bone anterior to the premolars has also been destroyed, leaving the partial roots of these teeth exposed. The alveolar bone around the right molars is well-preserved. Based on observation of the specimen and CT scans of the mandible (Figs. 2, S5), the right M_1_ root is present in the socket. The right M_2_ root and the medial side of the M_2_ crown are preserved and the right M_3_ root and crown are complete. The roots of the right M_3_ are highly bifurcating, indicating a nontaurodont molar. It is also observed on CT scan that the apices of the roots are lingual to the vertical axis of the teeth such that the roots are directed bucally rather than vertically. On the left side, the alveolar bone around the canine and the buccal side of the P_3_ is heavily damaged. The left P_4_ alveolus is intact but the tooth is missing. The left molar sockets are intact, and the left molars retain partial roots within their sockets but no tooth crowns are preserved.

1. Symphysis

Due to post-mortem damage, the superior symphysis is unobservable; only the inferior half of the symphysis is visible (ca. 16.4 mm from the basal margin). There is damage to the labial I_1_-I_1_ interdental septum, but the alveolar border lateral to the left C_1_ is present, giving a good approximation of its height (30.0 mm).

In profile, the greatest projection of the symphysis occurs at the base of the mandible. Along the right and left sides of the midline, it is possible to see a slight concavity below the alveolar plane, forming an *incurvatio mandibulae*. The TPL2 mandible has a raised, midline *tuber symphyseos* covering the inferior half of the symphysis. The superior-most aspect of the *tuber symphyseos* begins as a narrow ridge measuring 5.0 mm in width ca. 13.0 mm inferior to the (estimated) alveolar border. On the inferior symphysis, the *tuber symphyseos* becomes prominent with pogonion identified approximately 22.0 mm below the alveolar border. Very shallow depressions are located lateral to the *tuber symphyseos* (immediately lateral to the superior, narrow ridge). Paired lateral tubercles (*tuberculum laterale*) form the corners of a broad trigone, rounding onto the lateral and inferior body below the P_1_ on the right and left sides. The breadth of the lateral tubercles is approximately 39.0 mm. The mentum osseum rank of TPL2 is category 4 ([38](#_ENREF_38)) given its clear mentum osseum and lateral tubercles that are broad but without significant projection along the inferior border.

3. Lateral corpus

In *norma lateralis*, the basal and alveolar margins on the left side are roughly parallel. This is indicated by the corpus height measurements from C_1_-P_3_ to M_2_-M_3_, which vary by less than 1.0 mm (29.2 mm and 28.6 mm, respectively). On the right side, however, the height of the corpus decreases distally. At the C_1_-P_3_, the corpus measures 31.9 mm in height, but at the M_2_-M_3_ the height is only 25.1 mm, a difference of more than 6 mm. On each side, the superior lateral torus is poorly defined anteriorly but transitions abruptly into a highly developed lateral prominence with an apex located inferior to the M_2_-M_3_. This position is more anterior than in archaic human samples where it is usually located under the M_3_. The apex of the prominence on the right side is ca. 13.6 mm above the basal margin, and it is much more pronounced than the swelling on the left side. On the left, the apex of the prominence is located ca. 13.0 mm above the basal margin. The anterior marginal tubercles are present bilaterally. Their position is mesial, similar to what is found in all human groups except in Neandertals where the position is more distal ([39](#_ENREF_39)).

There is a single mental foramen on each side of the mandible, and each is located below P_4_-M_1_. The right mental foramen is positioned 14.6 mm inferior to the alveolar margin and 11 mm superior to the basal margin. It is oval in shape, oriented parallel to the oblique line and 5.0 mm x 3.7 mm in size. The left mental foramen is located 14.5 mm inferior to the alveolar margin and 13.2 mm superior to the basal margin. It is also oval in shape and parallel to the oblique line and 4.8 mm x 3.4 mm in size. In early modern humans, the mental foramen tends to have a mesial position (below P_4_) relative to a more distal position in archaic humans (below P_4_/M_1_ or M_1_) (Table S3). Although this is the “more archaic” condition, the position of the foramen in TPL2 is the same as that found in the majority of East Asian early modern humans (61.1%) (Table S3) and is most likely a reflection of the very short mandibular length of the individual ([40](#_ENREF_40), [41](#_ENREF_41)).

On the lingual side of the lateral corpus, the expression of the mylohyoid line is moderate, beginning 9 mm inferior to the distal edge of the M_3_ alveolus and running anteroinferiorly to a point 20 mm below the mesial edge of the M_1_ alveolus. The alveolar border shows a thickening from the M_1_-M_2_ (20.5 mm) to the M_3_ (18 mm), forming a mandibular torus below the alveolar process. The subalveolar plane increases rapidly towards the anterior portion of the mandible, forming a *planum alveolare* that extends from below the M_1_ towards the symphysis. The mesial endomandibular thickness is at its highest value at the M_1_ level (19 mm) and decreases towards the M_3_. There are bilaterally large digastric fossae and submaxillary fossae but no sublingual fossae.

4. Ramus

The lateral face of each ramus exhibits a more or less flat *fossa masseterica*, introverted gonion and well-developed *torus triangularis*. The minimum ramus breadths for TPL2 - 34.4 mm and 35.9 mm for the right and left rami, respectively - are low for an early modern human (Table 3).

On the right ramus, both the coronoid process and the condyle are missing at the approximate level of the mandibular notch, making an estimate of its position unreliable. The left superior ramus is better preserved with a complete coronoid process but without a condyle. The left mandibular notch is shallow with an estimated depth of 3.5 mm when measured relative to the coronoid process. The lowest point of the TPL2 mandibular notch lacks its posterior end, but its anterior and middle parts are in a position just posterior to the coronoid process; its lowest point is located anterior to the mandibular foramen, assuring that the notch was symmetrical. Archaic and early modern humans are differentiated by the positioning of the mandibular notch, and TPL2 is similar to the majority of early modern humans in having a symmetrical notch where the lowest point is approximately midway between the coronoid process and the mandibular condyle (Table S3). In contrast, the majority of archaic humans demonstrate an asymmetric notch with the lowest point shifted posteriorly to a position just anterior to the neck of the condyle^31^. Similar to the archaic condition, three earlier (Middle Pleistocene) East Asian partial mandibles from Zhoukoudian Locality 1 also have asymmetrical notches ([42](#_ENREF_42)).

The gonial regions are evenly rounded, and they curve slightly medially at gonion (along the posteroinferior margin of the ramus). There is no ante-gonial notch, the absence of which is common among Late Pleistocene humans ([43](#_ENREF_43)). The left masseteric insertion is weakly marked; on the right side, there is one large tubercle along the inferior margin of the masseteric insertion. There are relatively large tuberosities for the medial pterygoid muscles on the medial posteroinferior rami, as is often found among Neandertals and eastern Late Pleistocene humans, and these are more pronounced and extensive on the right side than on the left.

5. Dental crowns

Only the M_3_ crown is complete, and the occlusal surface exhibits a typical five-cusp pattern. The usual helicoidal wear typical of human molars is present, and it shows an ASUDAS wear score of 2. The crown is small with a buccolingual (BL) diameter of 10.8 mm and a mesiodistal (MD) diameter of 11.0 mm. This is within the range of other East Asian early modern humans (10.3 mm ± 0.5, n=34 for BL diameter and 10.6 mm ± 0.7, n=34 for MD diameter), although there is little difference among archaic and early modern human samples in M_3_ crown diameters ([43](#_ENREF_43)).

The Rodent faunal list

The rodent material from Tam Pa Ling comes from the TPL trench between 0.74 and 4.5 m depth. It consists of 366 cranial and dental remains. Of these, 117 were unidentifiable because of the lack of teeth. The taxonomy follows that of Wilson and Reeder ([44](#_ENREF_44)). In this preliminary study, among the 249 identified specimens (Table S9, NISP), some have been identified to the Family level (1 specimen of Hystricidae and 1 specimen of Sciuridae) or to the Sub-Family level (3 specimens of Rhizomyinae, 10 specimens of Arvicolinae and 27 specimens of Murinae (small, medium and large sizes)). The majority has been identified to the genus level: cf. *Bandicota* sp. (1 specimen), *Hapalomys* sp. (1 specimen), *Chiropodomys* sp. (1 specimen), *Mus* sp. (2 specimens), cf. *Berylmys* sp. (3 specimens), *Rattus* sp. (34 specimens) and *Niviventer* sp. (65 specimens). It was possible to identify specifically two taxa: *Belomys pearsonii* (8 specimens) and *Leopoldamys* cf. *sabanus* (92 specimens).

This fauna is dominated by three taxa, *L.* cf. *sabanus*, *Niviventer* sp. and *Rattus* sp. representing about 77% of all the identifiable material. At this stage of the study, all of the taxa identified are extant, so no biochronological inferences can be made.

According to Corbet and Hill ([45](#_ENREF_45)) and ([46](#_ENREF_46)), all of the taxa are still present today in the Tam Pa Ling region, except *Belomys pearsonii* and the Arvicolinae. *Belomys pearsonii* has a wide but patchy distribution in Southeast Asia. It lives in forests as well as *L.* cf. *sabanus*, *Niviventer* sp., *Berylmys* sp., *Chiropodomys* sp. or *Hapalomys* sp. Forest is definitely the main component indicated by the rodent fauna at Tam Pa Ling. The arvicolines are not found any more today in the region. They might indicate some cooler environmental component. The species determination of these arvicolines is currently under study.

1. Supplementary References

1. Arambourg C, Fromaget J. Le gisement quaternaire de Tam Hang (Chaine Annamitique septentrionale). Sa stratigraphique et ses faunes. C R Acad Sc Paris.1938; 203: 793-795.

2. Fromaget J. La stratigraphie des dépôts préhistoriques de Tam Hang (Chaîne Annamitique septentrionale) et ses difficultés. Proceedings of the Third Congress of Prehistorians of the Far East, Singapore 1938; 1940. pp. 60-70.

3. Bacon AM, Demeter F, Tougard C, De Vos J, Sayavongkhamdy T, Antoine PO, et al. The discovery of a Pleistocene fauna in karstic fills at Tam Hang in Laos: preliminary results. C R Palevol, 2008; 7(5): 277-288.

4. Bacon AM, Duringer Ph, Antoine PO, Demeter F, Shackelford L, Sayavongkhamdy T, et al. The Middle Pleistocene mammalian fauna from Tam Hang karstic deposit, northern Laos: New data and evolutionary hypothesis. Quat Int. 2011; 245(2): 315-332.

5. Duringer P, Bacon A-M, Sayavongkhamdy T, Nguyen TKT. Karst development, breccias history and mammalian assemblages in Southeast Asia: a brief review. C R Palevol. 2012; 11: 133-157.

6. Bacon AM, Demeter F, Duringer Ph, Patole-Edoumba E, Sayavongkhamdy T, Coupey AS, et al. Les sites de Tam Hang, Nam Lot et Tam Pa Ling au nord du Laos. Des Gisements à vertébrés aux origines des Hommes modernes. Editions CNRS, Paris; 2012.

7. Fromaget J. Sur la stratigraphie des formations récentes de la Chaîne annamitique septentrionale et sur l’existence de l’Homme dans le Quaternaire inférieur de cette partie de l’Indochine. C. R. Acad. Sci., Paris 1936; 203: 738-741.

8. Fromaget J. Aperçu sur la Stratigraphie et l’Anthropologie préhistorique des formations récentes dans la Chaîne Annamitique et le Haut-Laos. Compte rendu de la douzième session du Congrès préhistorique de France, Toulouse-Foix; 1936. pp.785-798.

9. Demeter F, Shackelford L, Westaway KE, Duringer Ph, Sayavongkhamdy T, Bacon AM. Reply to Pierret et al.: Stratigraphic and dating consistency reinforces the status of Tam Pa Ling fossil. Proc Natl Acad Sci U S A. 2012; 109(51):E3524-E3525.

10. Shackelford LL, Demeter F. The place of Tam Hang in Southeast Asian human evolution. C R Palevo. 2012; 11: 97-115.

11. Demeter F, Shackelford L, Bacon AM, Duringer Ph, Westaway K, Sayavongkhamdy T, et al. Anatomically modern human in Southeast Asia (Laos) by 46 ka. Proc Natl Acad Sci U S A. 2012; 109(36): 14375-14380.

12. Pierret A, Zeitoun V, & Forestier H. Irreconcilable differences between stratigraphy and direct dating cast doubts upon the status of Tam Pa Ling fossil. Proc Natl Acad Sci U S A. 2012; 109(51): E3523-E3523.

13. Aitken MJ. Thermoluminescence Dating. Academic Press, London; 1985.

14. Aitken MJ. An Introduction to Optical Dating. Oxford University Press, Oxford; 1998.

15. Huntley DJ, Godfreysmith DI, Thewalt MLW. Optical dating of sediments. Nature. 1985; 313(5998): 105-107.

16. Botter-Jensen L, Bulur E, Duller GAT, Murray AS. Advances in luminescence instrument systems. Radiat Meas. 2000; 32(5-6): 523-528.

17. Botter-Jensen L, Andersen CE, Duller GAT, Murray AS. Developments in radiation, stimulation and observation facilities in luminescence measurements. Radiat Meas. 2003; 37(4-5): 535-541.

18. Murray AS, Wintle AG. Luminescence dating of quartz using an improved single-aliquot regenerative-dose protocol. Radiat Meas. 2000; 32(1): 57-73.

19. Olley JM, De Deckker P, Roberts RG, Fifield LK, Yoshida H, Hancock G. Optical dating of deep-sea sediments using single grains of quartz: a comparison with radiocarbon. Sediment Geol. 2004; 169(3-4): 175-189.

20. Olley JM, Roberts RG, Yoshida H, & Bowler JM. Single-grain optical dating of grave-infill associated with human burials at Lake Mungo, Australia. Quat Sci Rev. 2006; 25(19-20): 2469-2474.

21. Wintle AG, Murray AS. A review of quartz optically stimulated luminescence characteristics and their relevance in single-aliquot regeneration dating protocols. Radiat Meas. 2006; 41: 369-391.

22. Duller GAT. Distinguishing quartz and feldspar in single grain luminescence measurements. Radiat Meas. 2003; 37: 161-165.

23. Jacobs Z, Duller GAT, Wintle AG. Interpretation of single grain D-e distributions and calculation of D-e. Radiat Meas. 2006; 41(3): 264-277.

24. Yoshida H, Roberts RG, Olley JM, Laslett GM, Galbraith RF. Extending the age range of optical dating using single 'supergrains' of quartz. Radiat Meas. 2000; 32(5-6): 439-446.

25. Duller GAT. Luminescence dating of Quaternary sediments: recent advances. Journal of Quat Sci. 2004; 19(2):183-192.

26. Galbraith RF, Roberts RG, Laslett GM, Yoshida H, Olley JM. Optical dating of single and multiple grains of quartz from jinmium rock shelter, northern Australia, part 1, Experimental design and statistical models. Archaeometry. 1999; 41: 339-364.

27. Roberts R, Bird M, Olley J, Galbraith R, Lawson E, Laslett G, et al. Optical and radiocarbon dating at Jinmium rock shelter in northern Australia. Nature. 1998; 393(6683): 358-362.

28. Stokes S, Ingram S, Aitken MJ, Sirocko F, Anderson R, Leuschner D. Alternative chronologies for Late Quaternary (Last Interglacial-Holocene) deep sea sediments via optical dating of silt-sized quartz. Quat Sci Rev. 2003; 22(8-9): 925-941.

29. Mejdahl V. Thermoluminescence dating: beta-dose attenuation in quartz grains. Archaeometry. 1979; 21: 61-72.

30. Feathers JK, Migliorini E. Luminescence dating at Katanda - a reassessment. Quat Sci Rev. 2001; 20(5-9): 961-966.

31. Jacobs Z. Development of luminescence techniques for dating Middle Stone Age sites in South Africa. Unpublished Ph.D. Thesis. University of Wales, Aberystwyth; 2004.

32. Prescott JR, Hutton JT. Cosmic-ray contributions to dose-rates for luminescence and ESR dating - large depths and long-term time variations. Radiat Meas. 1994; 23(2-3): 497-500.

33. Southon JR. Graphite reactor memory - where is it from and how to minimize it? Nucl Instrum Methods Phys Res B. 2007; 259(1): 288-292.

34. Stuiver M, Polach HA. Discussion: Reporting of ^14^C data. Radiocarbon. 1977; 19: 5-363.

35. Reimer PJ, Bard E, Bayliss A, Beck JW, Blackwell PG, Bronk Ramsey C, et al. IntCal13 and Marine13 radiocarbon age calibration curves 0–50,000 years cal BP. Radiocarbon. 2013; 55(4): 1869-1887.

36. Olley JM, Murray A, Roberts RG. The effects of disequilibria in the uranium and thorium decay chains on burial dose rates in fluvial sediments. Quat Sci Rev. 1996; 15(7):751-760.

37. Olley JM, Roberts RG, Murray AS. Disequilibria in the uranium decay series in sedimentary deposits at Allen's Cave, Nullarbor Plain, Australia: Implications for dose rate determinations. Radiat Meas. 1997; 27(2): 433-443.

38. Dobson SD, Trinkaus E. Cross-sectional geometry and morphology of the mandibular symphysis in Middle and Late Pleistocene *Homo*. J Hum Evol. 2002; 43(1): 67-87.

39. Rosas A. Occurrence of neanderthal features in mandibles from the Atapuerca-SH site. Am J Phys Anthropol. 2001; 114(1): 74-91.

40. Quam RM, Smith FH. A reassessment of the Tabun C2 mandible. In: Takeru A, Aoki A, Bar-Yosef O, editors, Neanderthals and Modern Humans in West Asia. Plenum Press, New York; 1998. pp. 405-421.41. Trinkaus E (2006) Modern human versus Neandertal evolutionary distinctiveness. Current Anthropology 47(4):597-620.

42. Weidenreich F. The mandibles of *Sinanthropus pekinensis*. A comparative study. Palaeontologica Sinica. 1936; 7D: 1-162.

43. Shang H, Trinkaus E. The early modern human from Tianyuan Cave, China. Texas A&M University, College Station; 2010.

44. Wilson DE, Reeder DM. Mammal species of the world: a taxonomic and geographic reference. Johns Hopkins University Press, Baltimore, MD; 2005..

45. Corbet GB, Hill JE. The mammals of the Indomalayan region: a systematic review. Oxford University Press, New York; 1992.

46. IUCN (2014) The IUCN red list of threatened species. Version 2014.2. p <http://www.iucnredlist.org>.

47. Bräuer G. Osteometrie. Anthropologie, Knussmann R editor (G. Fischer, Stuttgart); 1988. pp. 160-232.

48. Nicholson E, Harvati K. Quantitative analysis of human mandibular shape using three-dimensional geometric morphometrics. Am J Phys Anthropol. 2006; 131(3): 368-383.

49. Viterbo KMA, Jungers W, Sutikna T, Saptomo EW, & Morwood M. 3D geometric morphometrics of the LB1 mandible support the new species diagnosis (*Homo* *floresiensis*). Am J Phys Anthropol. 2012; 147: 294-294.

50. Rosas A, Bastir M. Geometric morphometric analysis of allometric variation in the mandibular morphology of the hominids of Atapuerca, Sima de los Huesos site. Anat Rec A Discov Mol Cell Evol Biol. 2004; 278A(2): 551-560.
