# Supplementary figures and images for: Early Modern Humans and Morphological Variation in Southeast Asia: Fossil Evidence from Tam Pa Ling, Laos
Source: PLoS One. 2015 Apr 7;10(4):e0121193. doi: 10.1371/journal.pone.0121193 (PMC4388508; doi:10.1371/journal.pone.0121193)

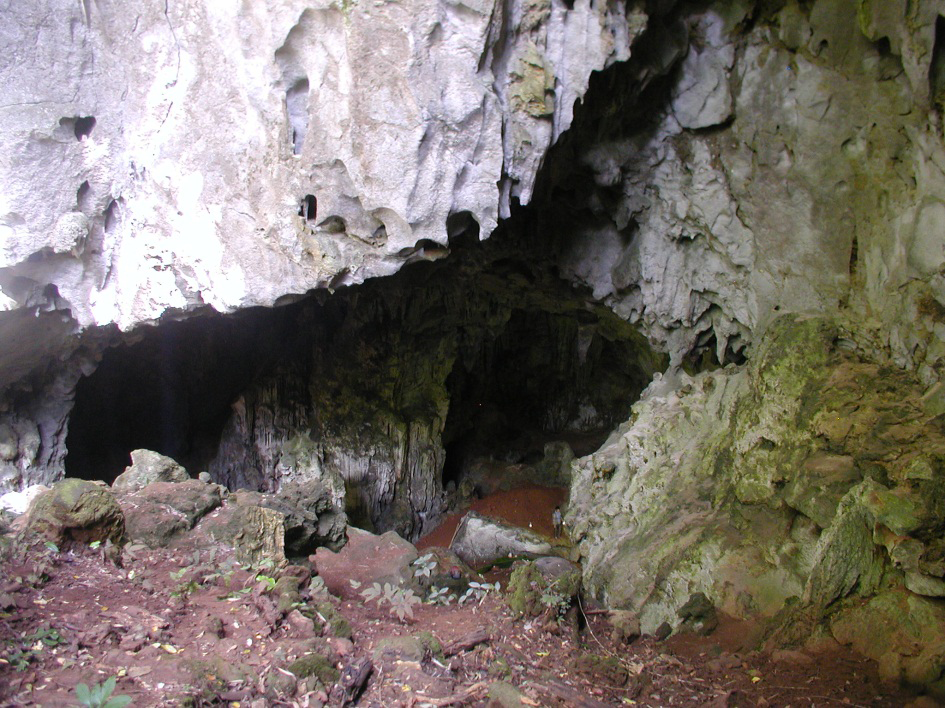

Supplement: S1 Fig — Photo taken from the south, looking towards the north. (TIF) [file pone.0121193.s003.tif]

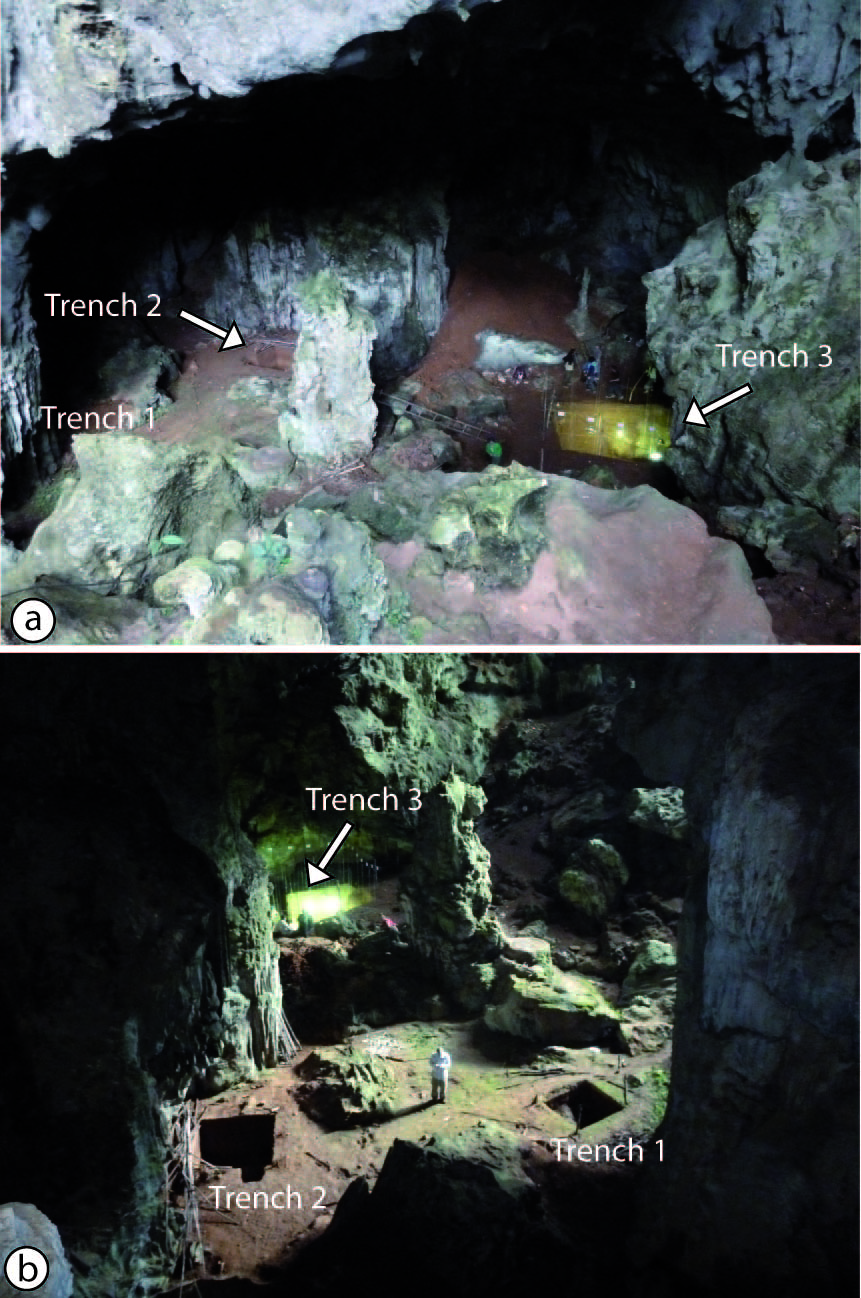

Supplement: S2 Fig — Top: Photo of the main gallery of TPL from the cave entrance, looking north. Test pits are shown on the left (trenches 1 and 2); excavation site is shown on the right (trench 3). Bottom: Photo of the main gallery of TPL from the west looking east. Test pits are shown in the foreground (trenches 1 and 2); excavation site is shown in the background (trench 3). (TIF) [file pone.0121193.s004.tif]

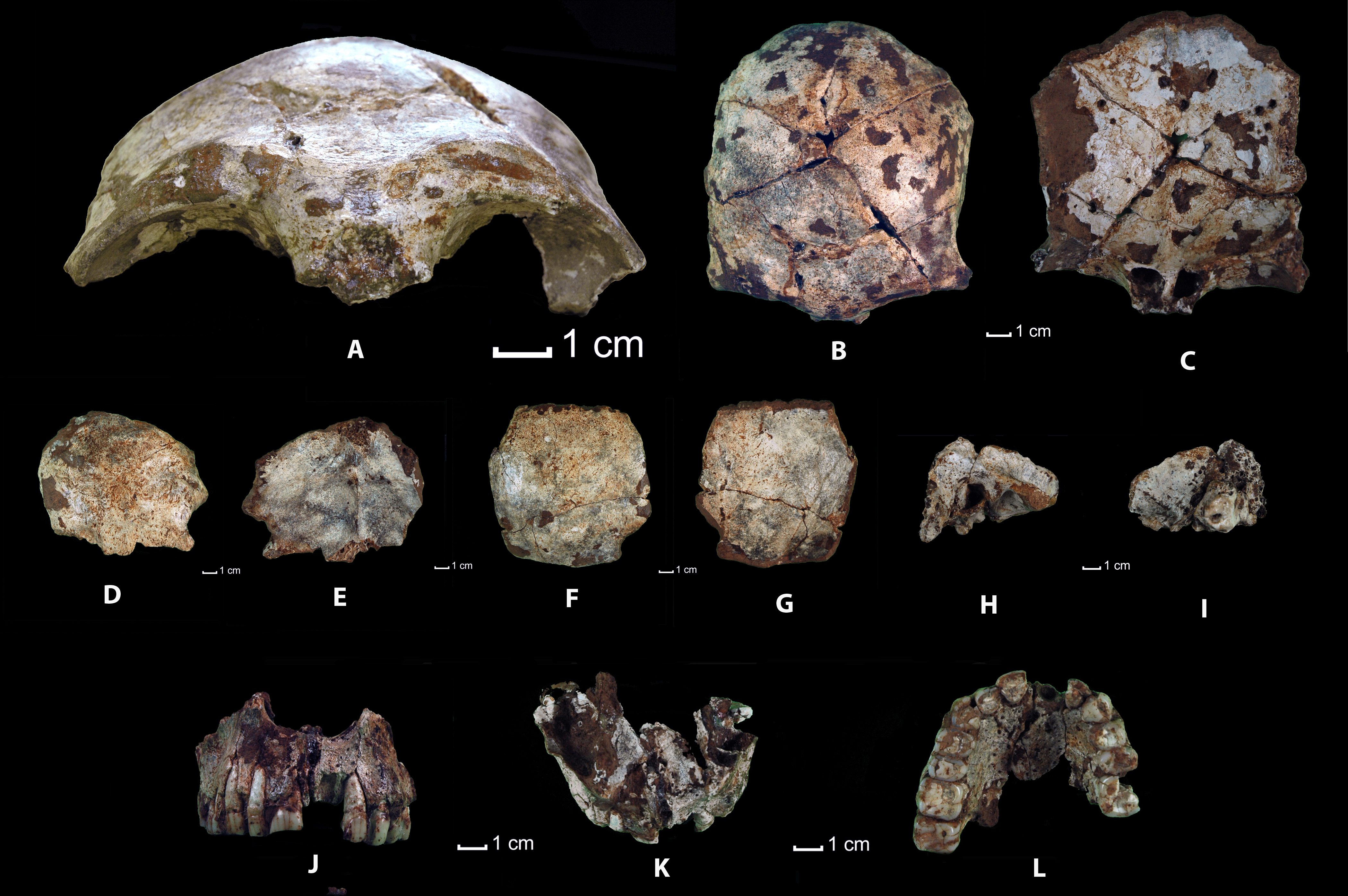

Supplement: S3 Fig — Elements: (A) frontal bone in norma facialis; (B) in norma verticalis; (C) in norma basilaris; (D) occipital bone in norma verticalis; (E) occipital bone in norma basilaris; (F) right parietal bone in norma verticalis; (G) right parietal bone in norma basilaris; (H) left temporal bone with partial mastoid in norma lateralis, external; (I) left temporal bone with partial mastoid in norma lateralis, internal; (J) maxillae in norma facialis; (K) maxillae in norma verticalis; (L) maxillae in norma basilaris. (TIF) [file pone.0121193.s005.tif]

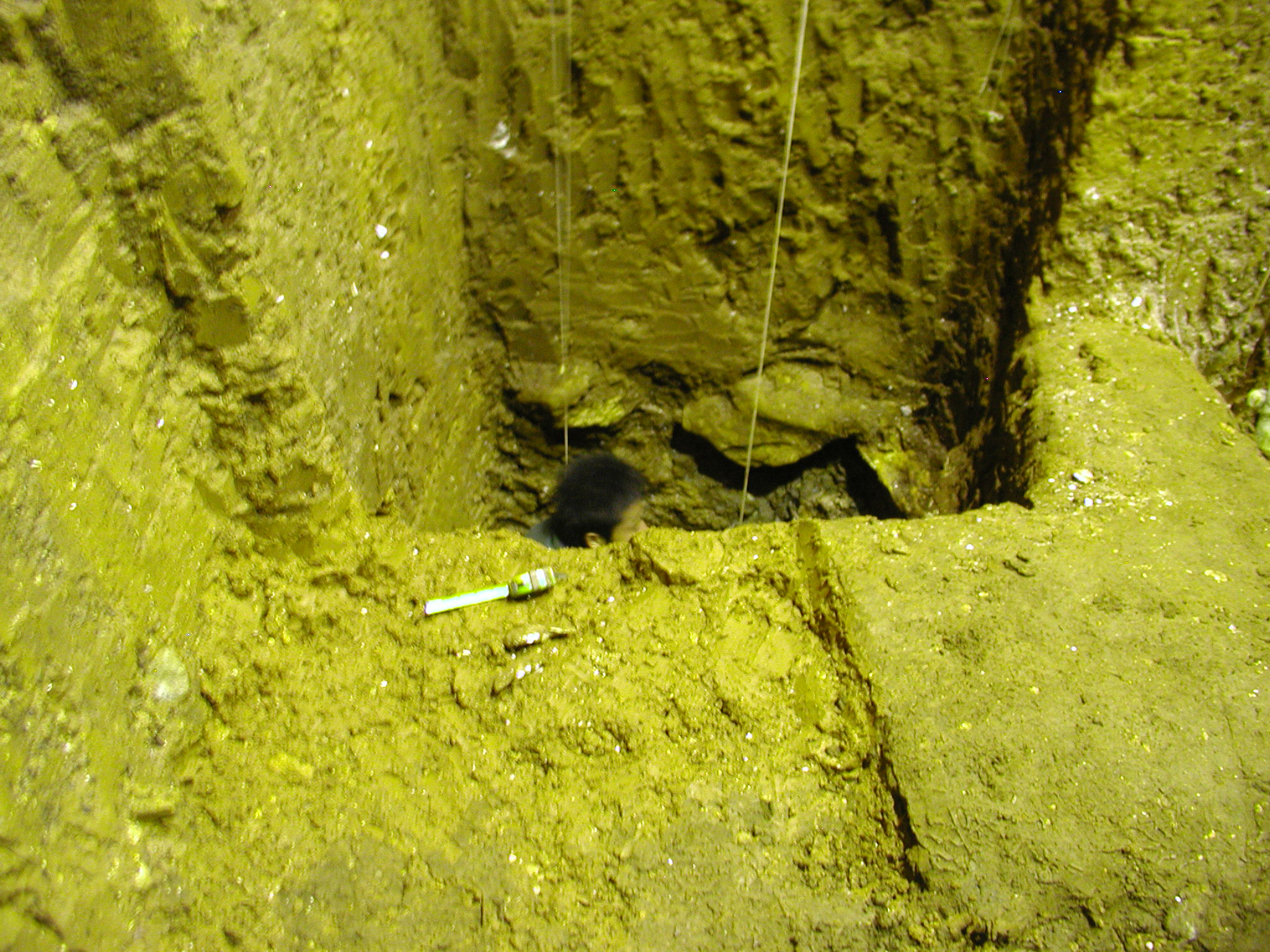

Supplement: S4 Fig — (TIF) [file pone.0121193.s006.tif]

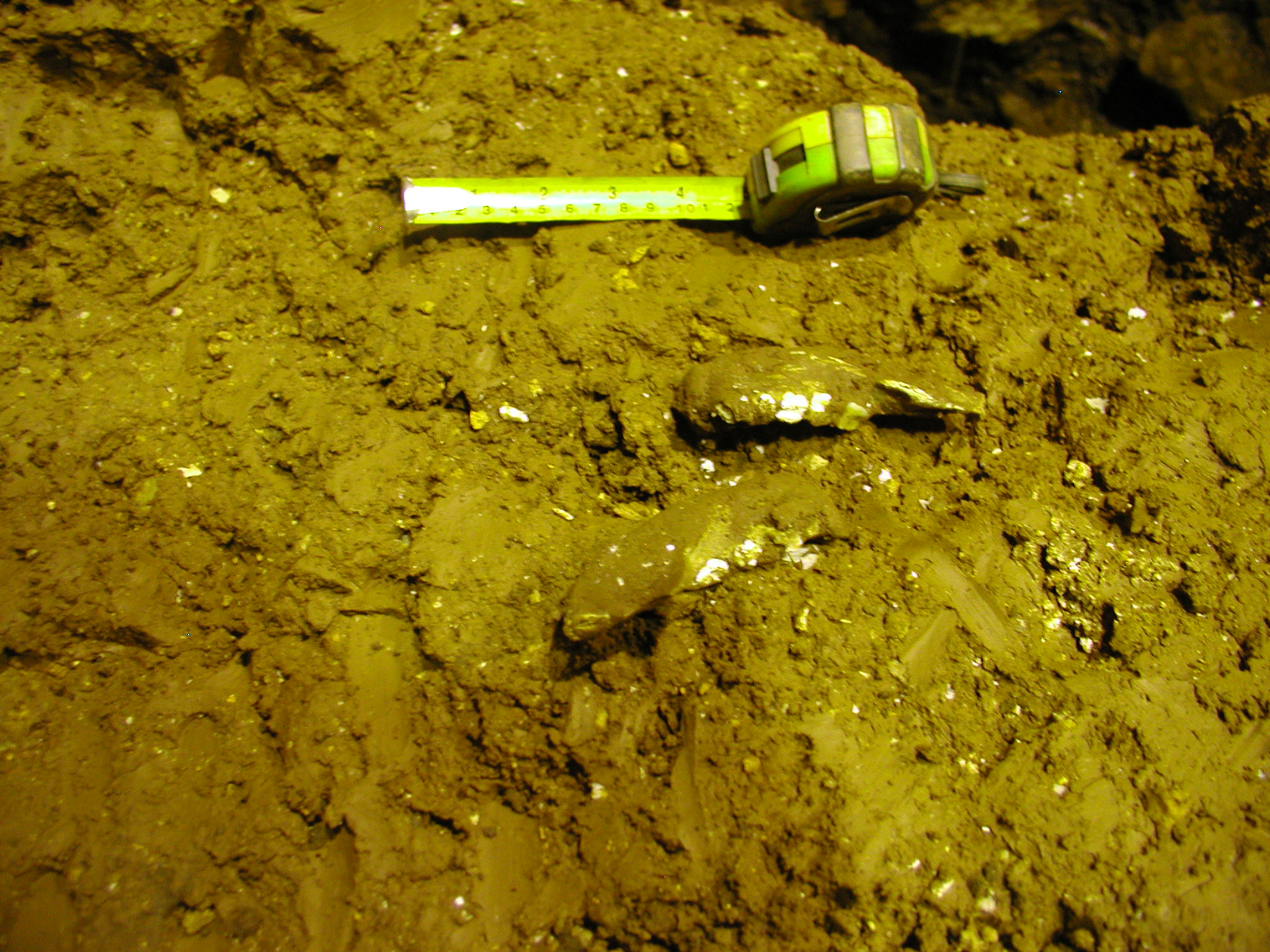

Supplement: S5 Fig — (TIF) [file pone.0121193.s007.tif]

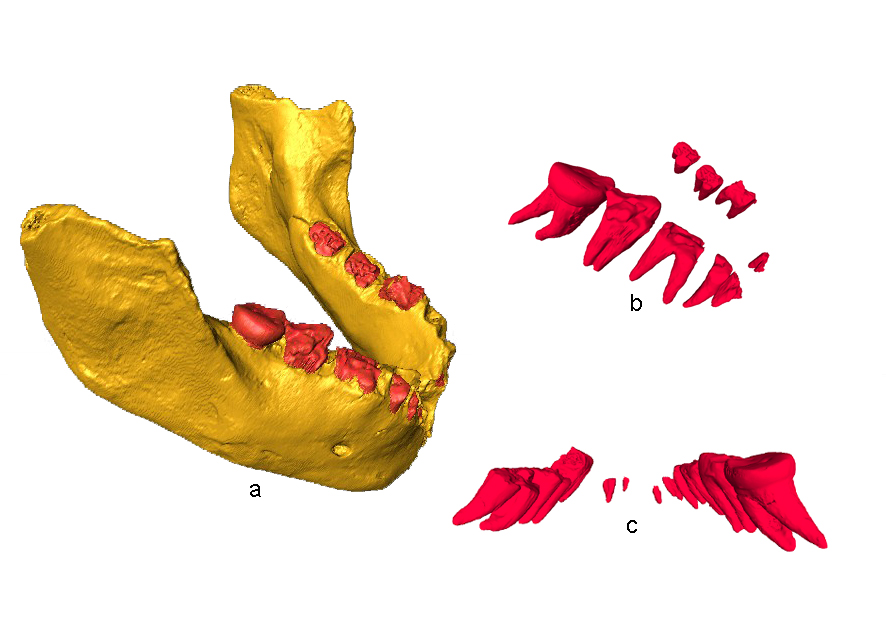

Supplement: S6 Fig — (a) the complete mandible; (b) the complete teeth segment extracted from the mandible corpus; (c) the complete teeth segment extracted in anteroposterior view. (TIF) [file pone.0121193.s008.tif]

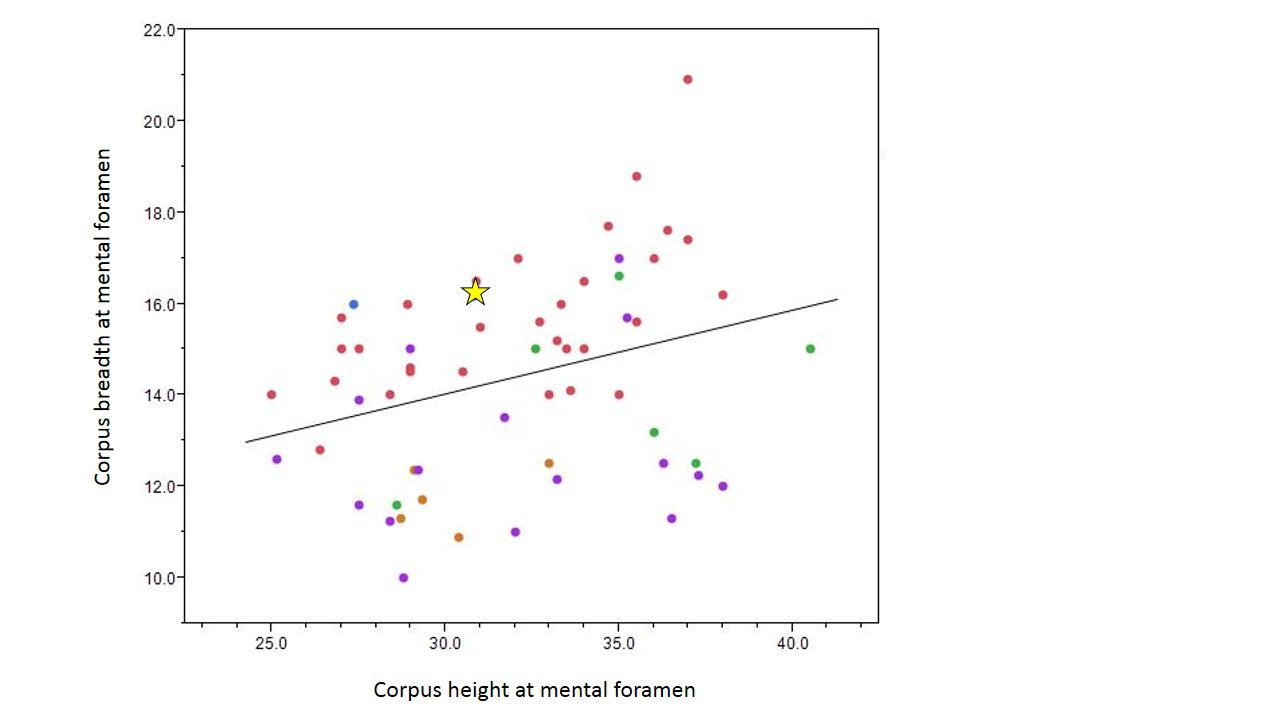

Supplement: S7 Fig — Red circles: Late Pleistocene archaic humans; green circles: Middle Paleolithic EMH; purple circles: western Eurasian EMH; orange circles: East Asian early modern humans; yellow star: TPL2. Black line is the linear regression line. (TIF) [file pone.0121193.s009.tif]

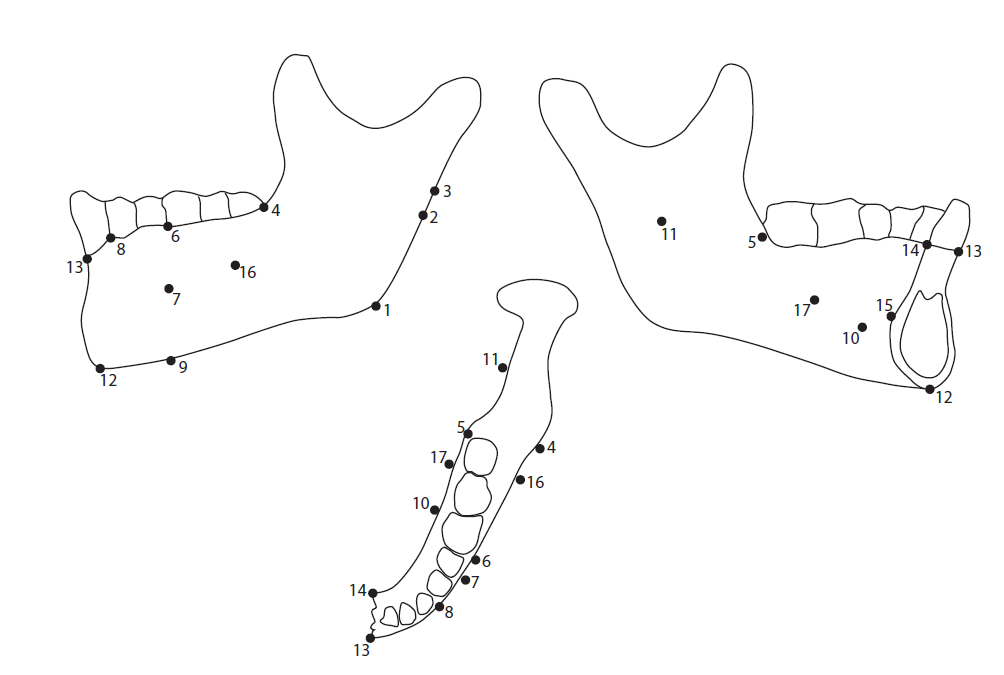

Supplement: S8 Fig — Drawing showing the position of the landmarks used for the geometric morphometrics analysis. (TIF) [file pone.0121193.s010.tif]

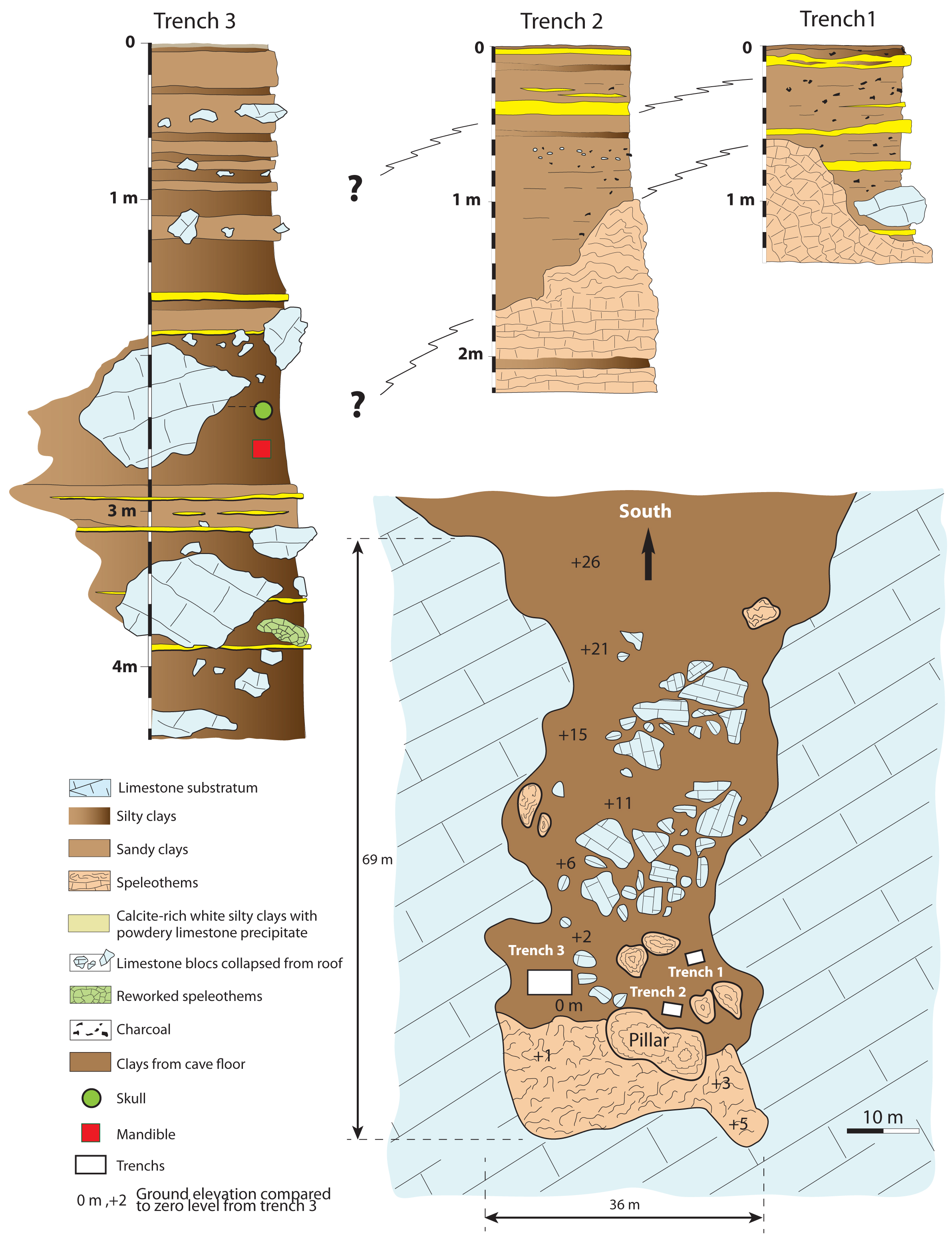

Supplement: S9 Fig — Stratigraphic sections from trenches 1–3 are shown and correlated. (TIF) [file pone.0121193.s011.tif]

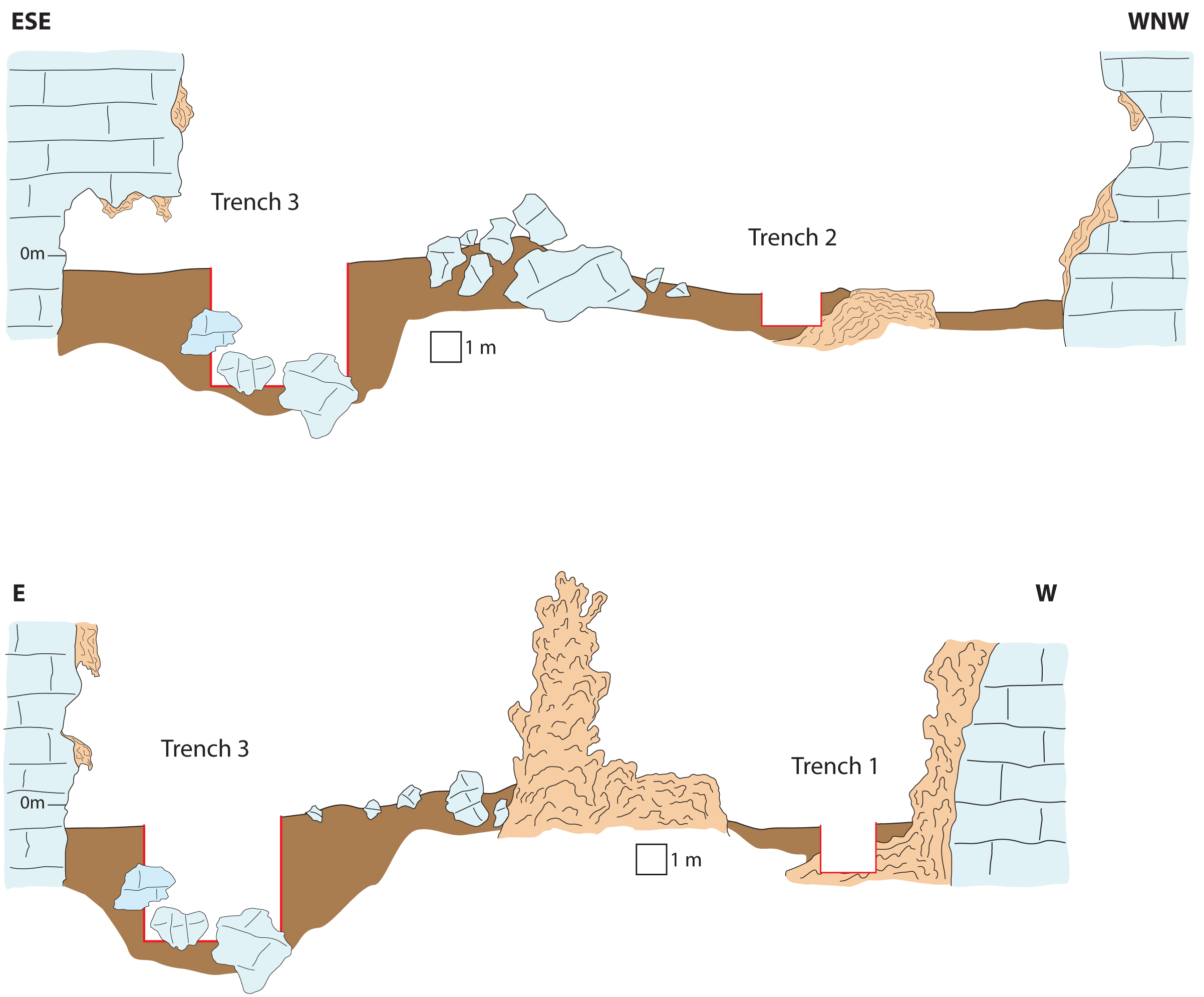

Supplement: S10 Fig — (TIF) [file pone.0121193.s012.tif]

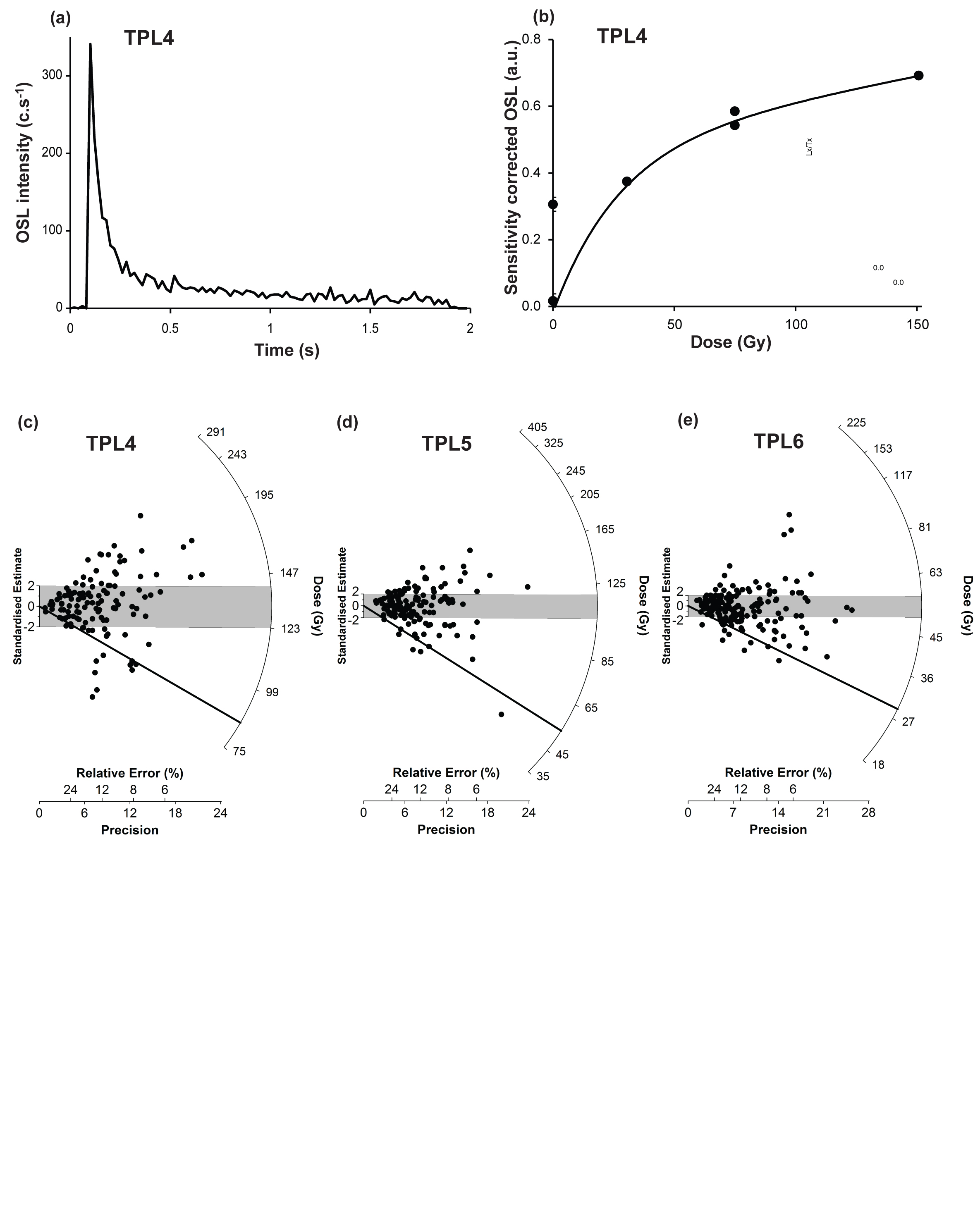

Supplement: S11 Fig — (a) representative shine down plot and (b) dose response curve is presented for sample TPL4-OSL. (c-g) single-grain distributions presented as radial plots for samples TPL4-OSL (c), TPL5-OSL (d), TPL6-OSL (e), TPL7-OSL (f), TPL8-OSL (g).The shaded area represents palaeodose values within 2Ïƒ of the central value for each distribution, while the solid line represents the palaeodose value determined using the minimum age model (MAM). (h) The relative profile likelihood for the MAM processed for sample TPL4-OSL displaying the optimization of the model and the generated palaeodose of 86 ± 5 ka. (TIF) [file pone.0121193.s013.tif]
