# Supplementary material for: Early Modern Humans and Morphological Variation in Southeast Asia: Fossil Evidence from Tam Pa Ling, Laos
Source: PLoS One. 2015 Apr 7;10(4):e0121193. doi: 10.1371/journal.pone.0121193 (PMC4388508; doi:10.1371/journal.pone.0121193)
Supplement: S1 Table — Measurements are in millimeters unless otherwise indicated. Estimated values are in parentheses. (DOCX) [file pone.0121193.s014.docx]

Table S1. TPL2 linear and angular dimensions, in millimeters unless otherwise indicated. Estimated values are in parentheses.

Right Left

Symphyseal height (M-69)^1^ 30.0

Symphyseal thickness 13.4

Anterior symphyseal angle (M-79(1b))² 68°

Gonial angle 121°

Mentum osseum rank^3^ 4

Mental foramen position P_4_-M_1_ P_4_-M_1_

Maximum length 77.0

Superior length (87.0)

Bigonial breadth 81.0

Bicondylar breadth (96.0)

Ramus length P_3_-M_3_ 44.5 44.4

Ramus height (39.0)

Ramus height at sigmoid notch 44.5 44.3

Ramus breadth 34.4 35.9

Corpus heights at:

C_1_ 32.4 29.6

C_1_/P_3_ 31.9 29.2

P_3_/P_4_ 29.6 30.2

M_1_/M_2_ 29.7 26.0

M_2_/M_3_  25.1 28.6

Mental foramen (M-69(1))^1^ 30.5 31.1

Corpus breadths at:

C_1_ 13.7 13.7

C_1_/P_3_ 14.8 14.7

P_3_/P_4_ 15.9 15.7

M_1_/M_2_ 18.3 18.6

M_2_/M_3_ 20.5 20.5

Mental foramen (M-69(3)) ^1^ 16.2 16.1

Mental foramen to alveolar border 11.0 10.1

______________________________________________________________________

^1^ M-#: Measurement definition in ([47](#_ENREF_47)).

^2^ Symphyseal angle oriented relative to the alveolar plane.
