# Supplementary material for: Early Modern Humans and Morphological Variation in Southeast Asia: Fossil Evidence from Tam Pa Ling, Laos
Source: PLoS One. 2015 Apr 7;10(4):e0121193. doi: 10.1371/journal.pone.0121193 (PMC4388508; doi:10.1371/journal.pone.0121193)
Supplement: S2 Table — (DOCX) [file pone.0121193.s015.docx]

Table S2. Discrete observations of the TPL2 mandibular corpus.

| **Discrete trait** | **Value** |
| --- | --- |
| Symphyseal profile in norma lateralis | Vertically-oriented or receding supero-posteriorly |
| Incurvatio mandibulae | Weakly developed |
| Tuber symphyseos | Moderately projecting |
| Central keel | Swelling on the anterior symphysis |
| Mental fossae | Shallow |
| Lateral tubercles | Observable but modest. Merge with the tuber symphyseos |
| Shelf-like anteroinferior symphysis | Absent |
| Anterior marginal tubercle | Absent |
| Superior lateral tubercle | Weakly developed |
| Incisura submentalis | Absent |
| Digastric fossae | Weak |
| Digastric fossae medial crest and spine | Absent |
| Alveolar planum | Absent |
| Genioglossal fossae | Weak |
| Superior transverse torus | Absent |
| Inferior transverse torus | Moderately developed |
| Extramolar sulcus | Wide |
| Retromolar space | Absent |
| Superior marginal torus | Poorly defined |
| Lateral prominence | Highly developed at M2-M3 |
| Trigonum mentale | Moderately developed |
