# Supplementary material for: Early Modern Humans and Morphological Variation in Southeast Asia: Fossil Evidence from Tam Pa Ling, Laos
Source: PLoS One. 2015 Apr 7;10(4):e0121193. doi: 10.1371/journal.pone.0121193 (PMC4388508; doi:10.1371/journal.pone.0121193)
Supplement: S3 Table — Comparative samples include Late Pleistocene (Late Pl.) archaic humans, Middle Paleolithic early modern humans (EMH) and Late Pleistocene early modern humans from East Asia (Late Pl. East Asian EMH) and western Eurasia (Late Pl. Western Eurasian EMH). (DOCX) [file pone.0121193.s016.docx]

Table S3. Discrete traits of the TPL2 mandible and their frequency in comparative samples. Comparative samples include Late Pleistocene (Late Pl.) archaic humans, Middle Paleolithic early modern humans (EMH) and Late Pleistocene early modern humans from East Asia (Late Pl. East Asian EMH) and western Eurasia (Late Pl. Western Eurasian EMH).

|  | Mentum osseum^1^  Rank %  (N) | Mental foramen position  Position %  (N) | Retromolar space  % absent (N) | Mandibular notch symm.  % present (N) | Mandibular foramen  % open (N) |
| --- | --- | --- | --- | --- | --- |
| TPL 2 | 4 | P_4_ (R)/M_1_ (L) | Absent | Present | Open |
| Late Pl. archaic humans | 1 11.5 %  2 57.7 %  3 30.8 %  (26) | P_4_  11.3 %  P_4_/M_1_  40.3 %  M_1_  48.4 %  (31) | 26.7 (30) | 26.7 (14) | 60.9 (23) |
| Late Pl. East Asian EMH | 4 56.3 %  5 43.8 %  (8) | P_4_  27.8 %  P_4_/M_1_  61.1 %  M_1_  11.1 %  (9) | 81.3 (8) | 100 (5) | 83.3 (6) |
| Late Pl. Western Eurasian EMH | 3 2.9 %  4 71.4 %  5 35.7 %  (35) | P_3_ 8.3%  P_4_  61.1%  P_4_/M_1_  22.2%  M_1_  8.3%  (36) | 85.0 (30) | 92.6 (27) | 97.9 (24) |

^1^ Mentum osseum ranked on a 1-5 scale following ^22^.
