# Supplementary material for: Early Modern Humans and Morphological Variation in Southeast Asia: Fossil Evidence from Tam Pa Ling, Laos
Source: PLoS One. 2015 Apr 7;10(4):e0121193. doi: 10.1371/journal.pone.0121193 (PMC4388508; doi:10.1371/journal.pone.0121193)
Supplement: S4 Table — (DOCX) [file pone.0121193.s017.docx]

Table S4. Landmarks taken on hemi-mandibles for geometric morphometric analysis.

|  | **Landmark** | **Definition** | **Reference** |
| --- | --- | --- | --- |
| 1 | Gonion | Point along rounded postero-inferior corner of mandible where line bisecting the angle between body and ramus would hit | ([48](#_ENREF_48)) |
| 2 | Alveolar-posterior ramus | Point at posterior margin of ramus at level of alveolar margin | ([49](#_ENREF_49)) |
| 3 | Posterior ramus | Point at posterior margin of ramus at level of M_3_ | ([48](#_ENREF_48)) |
| 4 | Root of anterior ramus | Point of anterior margin of the ramus that intersects with the corpus | ([49](#_ENREF_49)) |
| 5 | M_3_ | Point on alveolar bone just posterior to midline of third molar | ([48](#_ENREF_48)) |
| 6 | mesial M_1_ | Point on alveolar bone just mesial to M_1_ | ([50](#_ENREF_50)) |
| 7 | Mental foramen | Point in middle of mental foramen | ([48](#_ENREF_48)) |
| 8 | Canine | Point on alveolar margin between C and P_1_ | ([48](#_ENREF_48)) |
| 9 | mesial M_1_ on inferior border of corpus | Mesial M_1_ projected onto the inferior border of the corpus | ([49](#_ENREF_49)) |
| 10 | Mesial M_1_ on mylohyoid line | Mesial M_1_ projected onto mylohyoid line on the lingual aspect of the corpus | ([49](#_ENREF_49)) |
| 11 | Mandibular foramen | Most antero-inferior point of the mandibular foramen of the ramus | ([49](#_ENREF_49)) |
| 12 | Gnathion | Most inferior midline point on symphysis | ([48](#_ENREF_48)) |
| 13 | Infradentale | Midline point at superior tip of the septum between mandibular central incisors | ([48](#_ENREF_48)) |
| 14 | Mandibular orale | Most superior midline point on lingual side of mandible between two central incisors | ([48](#_ENREF_48)) |
| 15 | Superior transverse torus | Most posterior midline point on superior transverse torus | ([48](#_ENREF_48)) |
| 16 | Lingual M_2_-M_3_ corpus | Most distant point from lingual alveolar bone at M_2_-M_3_ |  |
| 17 | Buccal M_2_-M_3_ corpus | Most distant point from buccal alveolar bone at M_2_-M_3_ |  |
