# Supplementary material for: Early Modern Humans and Morphological Variation in Southeast Asia: Fossil Evidence from Tam Pa Ling, Laos
Source: PLoS One. 2015 Apr 7;10(4):e0121193. doi: 10.1371/journal.pone.0121193 (PMC4388508; doi:10.1371/journal.pone.0121193)
Supplement: S5 Table — Middle Pleistocene archaic and Holocene samples used only for geometric morphometric analysis. (DOCX) [file pone.0121193.s018.docx]

Table S5. Fossils included in comparative analyses. Middle Pleistocene archaic and Holocene samples used only for geometric morphometric analysis.

| **Archaic**  **humans (AH)** | | **Early modern humans (EMH)** | | | **Holocene humans** |
| --- | --- | --- | --- | --- | --- |
| **Middle Pleistocene AH** | **Late Pleistocene AH** | **Middle Paleolithic**  **EMH** | **Western Eurasian & African EMH** | **East Asian**  **EMH** |  |
| Arago 2, 13 | Amud 1 | Qafzeh 7, 8, 9, | Barma Grande 2 | Batatomba lena | F YNO 256 |
| Bodo cave 5 | Arcy-Hyène 9 | 10, 11, 15, 27 | Brno 2, 3 | Kow Swamp 5 | F YNO 273 |
| KNM LLA | Banyoles 1 | Skhul 4, 5, 6, 7 | Caviglione 1 | Liang Bua 1 | F YNO 274 |
| Montmaurin 1 |  |  | Combe Capelle | Minatogawa 1, A, 5, | F YNO 279 |
| Mauer | Combe Grenal 3 |  | Les Cottes 1 | 6, 7 | F YNO 305 |
| Pithecanthropus | La Ferrassie 1 |  | Cro-Mag 1(4253), | Moh Khiew 1 | F YNO 320 |
| 4 | Gegant |  | 3, 4256, 4258 | Tam Hang North 3 | F YNO 355 |
| Zhoukoudian | Guattari 2, 3 |  | Les Crouzade 4 | Tam Hang South 2, | F YNO271 |
| restored male | Kebara 2 |  | Dolní Věstonice 3, | 3, 4, 10, 11, 13, | M CO191 |
|  | Krapina 54-D, |  | 13, 14, 15, 16 | 13b, 13t, 14, 16, 22 | M YNO 260 |
|  | 55-E, 56-F, 58- |  | Fish Hoek | Tam Nang An | M YNO 261 |
|  | H, 59-J, 57-G |  | Grotte des Enfants 4 | Tam Pa Ling 2 | M YNO 292 |
|  | Malarnaud 1 |  | Isturitz | Tam Pong 1 | M YNO 297 |
|  | Moustier 1 |  | Kostenki 3, 4 | Tianyuan 1 | M YNO 309 |
|  | La Naulette 1 |  | Mitt Klause | Wadjak 2 | M YNO 311 |
|  | Palomas 1, 6, |  | Mladeč 52, 54, 56 | Zhoukoudian UC | M YNO 341 |
|  | 23, 59, 80 |  | Muierri 1 | 101, 103, 104, 108 | M YNO 343 |
|  | La Quina 5, 9 |  | Nazlet Khater 2 |  | M YNO268 |
|  | Regourdou 1 |  | Oase 1 |  | Modern 1345 |
|  | Saint-Césaire 1 |  | Ohalo 2 |  |  |
|  | Scladina 9, 4A-a |  | Paglicci 13, 14, 24, |  |  |
|  | Shanidar 1, 2, 4 |  | 25 |  |  |
|  | Sidron 1, 2 |  | Pataud 1 |  |  |
|  | Spy 1, 2 |  | Pavlov 1, 3 |  |  |
|  | Subalyuk 1 |  | Předmostí 1, 3, 4, 5, |  |  |
|  | Sveduy stul 1 |  | 9, 10, 14, 18, 21, |  |  |
|  | Tabun 1 |  | 24, 25, 26, 30 |  |  |
|  | Vindija 206, |  | QuinaAval 4 |  |  |
|  | 226, 231, 250 |  | Rois 1 |  |  |
|  | Zafarraya |  | Sunghir 1, 6 |  |  |
|  |  |  | Willendorf |  |  |
