# Supplementary material for: Early Modern Humans and Morphological Variation in Southeast Asia: Fossil Evidence from Tam Pa Ling, Laos
Source: PLoS One. 2015 Apr 7;10(4):e0121193. doi: 10.1371/journal.pone.0121193 (PMC4388508; doi:10.1371/journal.pone.0121193)
Supplement: S6 Table — (DOCX) [file pone.0121193.s019.docx]

Table S6. OSL single-grain rejections.

| **Sample** | **Processed**  **grains** | **Rejected**  **grains** | **Accepted**  **grains** | **Proportion of luminescence emitting grains** | **Rejection rate**  **(%)** | **Overdispersion**  **(%)** | **Statistical model** |
| --- | --- | --- | --- | --- | --- | --- | --- |
| TPL4-OSL | 900 | 779 | 121 | 13% | 87 | 32.5 | MAM |
| TPL5-OSL | 800 | 664 | 136 | 17% | 83 | 32.8 | MAM |
| TPL6-OSL | 900 | 722 | 178 | 20% | 80 | 40.0 | MAM |
| TPL7-OSL | 1400 | 1279 | 121 | 9% | 91 | 31.7 | MAM |
| TPL8-OSL | 1800 | 1699 | 101 | 6% | 94 | 45.4 | MAM |

| **Criteria** | **TPL4-OSL** | **TPL5-OSL** | **TPL6-OSL** | **TPL7-OSL** | **TPL8-OSL** |
| --- | --- | --- | --- | --- | --- |
| Signal <3σBG | 297 | 280 | 300 | 697 | 918 |
| Recycling Ratio >10% unity | 194 | 158 | 109 | 142 | 145 |
| IR ratio IR >2σ signal | 148 | 119 | 216 | 55 | 98 |
| Saturated grains | 98 | 85 | 82 | 91 | 86 |
| Supersaturating  grains | 37 | 17 | 4 | 52 | 9 |
| Recuperation >5% | 2 | 2 | 10 | 4 | 34 |
| No decay | 1 | 0 | 0 | 235 | 406 |
| Dominance of medium component | 2 | 3 | 1 | 3 | 3 |
| **Total** | **779** | **664** | **722** | **1279** | **1699** |
