# Supplementary material for: Early Modern Humans and Morphological Variation in Southeast Asia: Fossil Evidence from Tam Pa Ling, Laos
Source: PLoS One. 2015 Apr 7;10(4):e0121193. doi: 10.1371/journal.pone.0121193 (PMC4388508; doi:10.1371/journal.pone.0121193)
Supplement: S7 Table — (DOCX) [file pone.0121193.s020.docx]

**Table S7.** ^14^C age estimates

| **ISGS** | **Sample** | **Material** | **δ^13^C** | **^14^C yr BP** | **± (1σ)** | **Cal. BP** | **± (1σ)** |
| --- | --- | --- | --- | --- | --- | --- | --- |
| A2657 | TPL PC-A | Charcoal | -25.3 | 34660 | 340 | 39155 | 385 |
| A2658 | TPL PC-B | Charcoal | -23.3 | 32220 | 260 | 36100 | 280 |
| A2659 | TPL PC-C | Charcoal | -24.8 | 32370 | 250 | 36260 | 280 |
| A2660 | TPL PC-D | Charcoal | -24.9 | 20870 | 70 | 25205 | 120 |

The half-life of 5568 is used for the age calculation. It is reported as BP (before present defined as before 1950). MC-Modern Carbon.
