# Supplementary material for: Early Modern Humans and Morphological Variation in Southeast Asia: Fossil Evidence from Tam Pa Ling, Laos
Source: PLoS One. 2015 Apr 7;10(4):e0121193. doi: 10.1371/journal.pone.0121193 (PMC4388508; doi:10.1371/journal.pone.0121193)
Supplement: S8 Table — Summary statistics provided as mean ± standard deviation, N. BL = buccolingual crown diameter; MD = mesiodistal crown diameter. Comparative samples as in S3 Table. (DOCX) [file pone.0121193.s021.docx]

Table S8. Mandibular molar crown diameters for TPL2 and comparative samples. Summary statistics provided as mean ± standard deviation, N. BL = buccolingual crown diameter; MD = mesiodistal crown diameter. Comparative samples as in Table S3.

| **Samples** | **M_3_ BL (mm)** | **M_3_ MD (mm)** | **M_3_ Area^1^ (mm^2^)** |
| --- | --- | --- | --- |
| TPL2 | 10.8 | 11.0 | 118.2 |
| Late Pl. East Asian EMH | 10.3 ± 0.5  34 | 10.6 ± 0.7  34 | 108.8 ± 10.4  34 |
| Late Pl. Western Eurasian EMH | 10.7 ± 0.8  30 | 11.3 ± 1.0  26 | 121.9 ± 19.4  26 |
| Middle Paleolithic EMH | 10.8 ± 0.8  7 | 11.9 ± 0.8  7 | 128.9 ± 15.5  7 |
| Late Pl. archaic humans | 11.0 ± 0.8  59 | 11.8 ± 0.7  56 | 129.8 ± 14.9  55 |
| Middle Pl. archaic humans | 10.9 ± 1.0  33 | 11.8 ± 1.2  33 | 129.3 ± 24.4  33 |

^1^ Area = MD x BL.
