# Supplementary material for: Early Modern Humans and Morphological Variation in Southeast Asia: Fossil Evidence from Tam Pa Ling, Laos
Source: PLoS One. 2015 Apr 7;10(4):e0121193. doi: 10.1371/journal.pone.0121193 (PMC4388508; doi:10.1371/journal.pone.0121193)
Supplement: S9 Table — On the 366 remains, 249 have been identified as shown in the table (NISP). The 27 remains of undetermined Murinae could be grouped as small, medium and large size murin, with the calculated MNI for each size. (DOCX) [file pone.0121193.s022.docx]

**Table S9.** Rodent faunal list for Tam Pa Ling. On the 366 remains, 249 have been identified as shown in the table (NISP). The 27 remains of undetermined Murinae could be grouped as small, medium and large size murin, with the calculated MNI for each size.

| **Taxon** | **Number of Identified specimens (NISP)** | **Minimum Number of Individuals (MNI)** |
| --- | --- | --- |
| Family Sciuridae indet.  Subfamily Sciurinae  *Belomys pearsonii*  Family Spalacidae  Subfamily Rhizomyinae indet.  Family Cricetidae  Subfamily Arvicolinae indet.  Family Muridae  Subfamily Murinae indet.  cf. *Bandicota* sp.  cf. *Berylmys* sp.  *Chiropodomys* sp.  *Hapalomys* sp.  *Leopoldamys* cf. *sabanus*  *Mus* p.  *Niviventer* sp.  *Rattus* sp.  Family Hystricidae indet. | 1  8  3  10  27  1  3  1  1  92  2  65  34  1 | 1  3  1  6  3 small, 7 medium, 3 large size  1  2  1  1  39  2  31  15  1 |
